# Manipulation of macrophage polarization by peptide-coated gold nanoparticles and its protective effects on acute lung injury

**DOI:** 10.1186/s12951-020-00593-7

**Published:** 2020-02-26

**Authors:** Lu Wang, Huasheng Zhang, Liya Sun, Wei Gao, Ye Xiong, Aying Ma, Xiali Liu, Lei Shen, Qiang Li, Hong Yang

**Affiliations:** 1 Department of Pulmonary and Critical Care Medicine, Shanghai General Hospital, Shanghai Jiao Tong University School of Medicine, Shanghai, 201620 China; 2 Shanghai Institute of Immunology, Shanghai Jiao Tong University School of Medicine, Shanghai, 200025 China; 3 School of Biomedical Engineering, Tianjin Medical University, Tianjin, 300070 China; 4 Department of Pulmonary and Critical Care Medicine, Shanghai East Hospital, Tongji University, Shanghai, 200120 China

**Keywords:** Peptide, Gold nanoparticle, Inflammation, Macrophage polarization, Alveolar macrophage, Acute lung injury

## Abstract

**Background:**

Macrophage polarization and reprogramming in the lung play a critical role in the initiation, development and progression of acute lung injury (ALI). Regulating the activation and differentiation of pulmonary macrophages may provide a potential therapeutic strategy to treat ALI. We previously developed a novel class of anti-inflammatory nanoparticles (P12) that can potently inhibit Toll-like receptor (TLR) signaling in macrophages. These bioactive nanodevices were made of gold nanoparticles (GNPs) coated with hexapeptides to not only ensure their physiological stability but also enable GNPs with TLR inhibitory activity.

**Results:**

In this study, using a lipopolysaccharide (LPS) induced ALI mouse model, we showed that P12 was able to alleviate lung inflammation and damage through reducing the infiltration of inflammatory cells and increasing the anti-inflammatory cytokine (IL-10) in the lung. These results prompted us to investigate possible macrophage polarization by P12. We first confirmed that P12 primarily targeted macrophages in the lung to exert anti-inflammatory activity. We then showed that P12 could drive the polarization of mouse bone marrow-derived macrophages (BMDMs) toward anti-inflammatory M2 phenotype. Interestingly, in the ALI mouse model, P12 was able to increase the alveolar M2 macrophages and reduce both the alveolar and interstitial M1 macrophages in the bronchoalveolar lavage fluid (BALF) and lung tissues.

**Conclusion:**

This study demonstrated that peptide-coated GNPs could induce M2 macrophage polarization in vitro and in vivo to effectively regulate lung inflammation, protect lung from injuries and promote inflammation resolution. The ability of regulating macrophage polarization together with TLR inhibition made such a bioactive nanodevice a new generation of potent therapeutics to treat ALI.
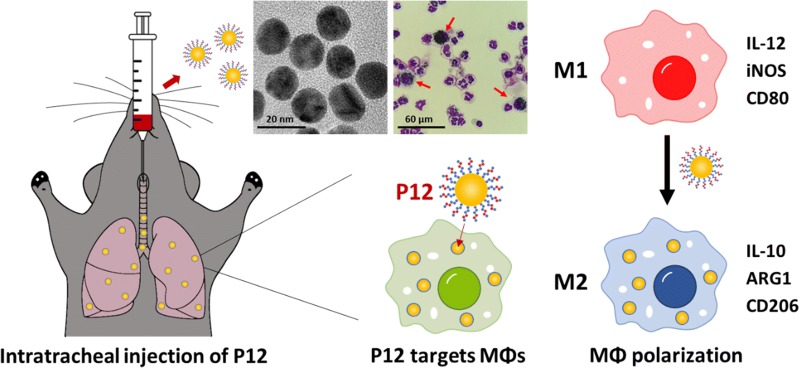

## Introduction

Acute lung injury (ALI) and its more severe form of acute respiratory distress syndrome (ARDS) belong to a spectrum of acute and life-threatening pulmonary inflammatory conditions with a mortality rate reaching 40% and above [1, 2]. The clinical manifestation is characterized by acute hypoxic respiratory failure caused by pulmonary (e.g., pneumonia and aspiration damage) and nonpulmonary (e.g., sepsis and trauma) insults [3]. The main pathogenic feature of such conditions is the overwhelming uncontrolled inflammatory reaction in the lung, such as cytokine storm and infiltration of inflammatory cells; this can lead to tissue damage and accumulation of proteinaceous edema in the pulmonary interstitial and alveolar spaces, causing overall acute, diffuse and inflammatory lung injuries [4]. To date, the only effective supportive therapy in clinics is the low tidal volume mechanical ventilation, which has shown about 10% reduction in mortality [5, 6]. Despite many efforts made to improve current treatments of ALI/ARDS, none of them are effective in clinical trials, and the mortality rate remains high [7]. Particularly, the development of new drugs to improve outcomes in ARDS patients is still relatively slow and under expectation. Therefore, it is urgent to develop new and potent pharmacological approaches to not only manage the intense acute inflammation, but also promote tissue repair in the lungs for ALI/ARDS patients.

The lung macrophages play a central role in the initiation and progression of inflammation in ALI/ARDS, and hence could be a potential therapeutic target for pharmacological interventions. They are viewed as the master immune cells governing both initiation and resolution phases of ALI/ARDS [8, 9]. Upon stimulation, resting macrophages (M0) are polarized into distinct functional phenotypes that can be mainly classified into the pro-inflammatory (classically activated, M1) and the anti-inflammatory (alternatively activated, M2) types according to different local microenvironments. In the early stage of ALI, the infection-triggered activation of Toll-like receptors (TLRs) or other pattern recognition receptors (PRRs) immediately shift resident alveolar macrophages (AMs) to the predominant M1 phenotype; M1 macrophages secrete large amount of pro-inflammatory cytokines to mount inflammatory responses and recruit neutrophils to the alveolar space [9, 10]. Thus, M1 macrophages serve as a driver in the process of lung inflammation contributing to the initiation of ALI/ARDS [11]. In the later resolution stage, lung macrophages can switch to anti-inflammatory/reparative M2 phenotype to accelerate resolution of inflammation and promote tissue repair. Therefore, macrophage polarization and reprogramming play an important role in the maintenance of immune homeostasis in the lung. The effective control of activation and differentiation of macrophages would help regulate the initiation, development and progression of lung injury, providing a potential therapeutic strategy to treat ALI/ARDS.

Macrophage polarization is intrinsically regulated by several known key transcription factors. They are nuclear factor-κB (NF-κB), signal transducer and activator of transcription (STAT) protein family, peroxisome proliferator-activated receptor gamma (PPAR-γ) and interferon regulatory factor (IRF) family [9, 12]. For example, studies have shown that M1 polarization involves the activation of STAT1 and IRF5, while M2 polarization is driven by STAT6/STAT3 and IRF4 activation. Based on these characteristics, various cytokines or cytokine cocktails have been applied to induce macrophages to differentiate into a specific phenotype. For example, IL-4 or IL-4 and IL-13 cocktail can induce M2 type [13], while LPS in the presence and absence of IFN-γ can induce M1 type macrophages [14, 15]. In addition, specific miRNAs or siRNAs have been designed to manipulate the expression of these transcription factors, allowing for reprograming macrophage polarization in various disease conditions [16, 17].

Nanodevices are capable of targeting macrophages simply because macrophages have superior phagocytosis ability to capture nanoscale particles [18, 19]. This is particularly advantageous for developing bioactive nanoparticles to control macrophage-mediated pathogenic inflammatory responses. Among various nanodevices, gold nanoparticles (GNPs) could be a promising platform to develop the next generation anti-inflammatory nanomedicine for the following reasons. First, GNPs can be easily synthesized, and their size can be precisely controlled from several to hundreds of nanometers. Second, their surface can be easily modified with peptides through the binding of the thiol group from the peptide (cysteine residue) to gold to alter the surface chemistry and impart new bioactivities. Third, GNP based nanodevices have a relatively safe profile in vitro and in vivo. In fact, a GNP-based nanodrug has been well tolerated in a phase I clinical trial of 30 advanced cancer patients [20], indicating that GNP based nanotherapeutics are potentially safe and translatable for future clinical uses.

For the past few years, we have developed a novel class of bioactive nanodevices that can inhibit multiple TLR signaling pathways of macrophages [21–23]. These nanodevices were made of GNPs coated with hexapeptides on the surface, which together make them bioactive (without carrying any drug) to effectively inhibit lipopolysaccharide (LPS) induced TLR4 activation in macrophages. Such peptide-GNP hybrids were able to reduce inflammation and prevent lung injuries in a LPS-induced ALI mouse model [24]. It was found that these hybrids were largely internalized into endosomal compartments in macrophages and exerted inhibitory activity by blocking the endosomal acidification [22]. Since these nanodevices primarily inhibited the activation of NF-κB and IRFs, we strongly suspected that they may also facilitate the polarization of macrophages toward anti-inflammatory M2 phenotype, contributing to the reduction of lung inflammation and promoting the resolution phase during ALI.

To test such a hypothesis, we herein systematically examined the effects of the peptide-coated GNPs (P12) on macrophage polarization in vitro and in vivo. The in vivo therapeutic activity and macrophage targeting capability of P12 were first demonstrated in a LPS-induced ALI mouse model. The important role of macrophages in the protective effects by the nanodevices was assessed using clodronate encapsulated liposomes to deplete alveolar macrophages. The effect of the nanodevice on macrophage polarization was examined using mouse bone marrow-derived macrophages (BMDMs) under polarization conditions (M1 or M2) in vitro. Finally, the phenotypes of alveolar and interstitial macrophages in the lung of the ALI mouse model were evaluated to confirm the action of the nanodevice on macrophage polarization. This study demonstrated a novel class of bioactive nanodevices that can reduce the inflammatory responses of M1 macrophages and promote macrophage polarization to the anti-inflammatory/reparative M2 phenotype, providing a new strategic approach to treat ALI/ARDS.

## Experimental

### Fabrication of peptide-coated GNPs and their physicochemical characterization

Gold nanoparticles (GNPs) were synthesized according to the literature and our earlier work [21, 25]. The peptide-coated GNPs (P12) were prepared following our previously published protocol [21, 22], where the hexapeptides (CLPFFD, from CanPeptide Inc., Montreal, Canada) at a concentration of 1 mM were mixed with GNP (11 nM) at a peptide-to-GNP volume ratio of 1:10. The fluorescent peptide-coated GNPs (P12-Cy5, 10 nM) was made by adding minute Cy5-PEG2000-SH (0.5%, from Nanocs Inc. New York, USA) to the peptide solution prior to mixing with the GNP solution. The mixtures were incubated at room temperature for various time periods, and the resulting P12 was analyzed by dynamic light scattering (DLS) technique (Zetasizer Nano ZS, Malvern Instruments, Worcestershire, UK) for its hydrodynamic diameter and tested in salt solutions for its physiological stability. For all cell culture and animal studies, P12 or P12-Cy5 was prepared by overnight incubation, followed by sterilization through filtration (0.22 µm, Milipore, Billerica, MA, USA) and 3-times washing with phosphate buffered saline (PBS) through centrifugation (15,000 rpm at 4 ℃ for 30 min) to remove free peptides; they were suspended in PBS or culture medium at required concentrations for further study.

The morphology and size of bare GNPs and peptide-coated GNPs were examined by the JEOL JEM-2100 electron microscope (Tokyo, Japan) at an accelerating voltage of 220 kV. DLS technique was conducted as a complement method to determine the hydrodynamic diameter of the nanoparticles. For stability analysis, the nanoparticles were mixed with the same volume of sodium chloride (NaCl) solution at various concentrations (0.3 M, 0.6 M and 1.5 M) or ultrapure water in a 96-well plate. After incubation for 2 h, their optical density (OD) was measured at 524 nm on a microplate reader (Bio-Rad, USA). The stability was assessed by the OD difference of the mixtures in water and NaCl solutions. All stability tests were performed at room temperature.

### Culture of BMDMs and macrophage polarization

Bone marrow cells were isolated from tibias and femurs of 8–12 weeks old C57BL/6 mice as previously described [26]. The cells were cultured in IMDM medium (Gibco, USA) supplemented with 10% FBS (Gibco, USA), 1% penicillin and streptomycin and 20 ng/mL M-CSF (PeproTech, USA), and derived into macrophages (BMDMs) over time. On day 3, fresh BMDM culture medium (half of original volume) was added. On day 7, macrophages were harvested to assess the purity of mature BMDMs by flow cytometry and plated at a density of 1 × 10^6^ cells/mL for further experiments.

For M1 polarization, mature BMDMs were co-stimulated with 10 ng/mL LPS (InvivoGen, USA) and 10 ng/mL IFN-γ (PeproTech, USA) for 24 h; for M2 polarization, they were alternatively co-stimulated with 10 ng/mL IL-4 (PeproTech, USA) and 10 ng/mL IL-13 (PeproTech, USA) for 24 h. In P12 intervention group, P12 treatment (12.5 nM or 50 nM) was applied together with the polarization stimuli. After 24 h, the culture media were collected and centrifuged at 2000 rpm for 10 min at 4 ℃ for cytokine analysis. Meanwhile, the total RNA of BMDMs was extracted for the analysis of M1 or M2 specific gene expression. The viability of BMDMs upon P12 treatment during M1 and M2 polarization was measured by MTS assay.

### LPS-induced ALI mouse model

Mice were purchased from Shanghai Laboratory Animal Co. Ltd. (Shanghai, China) and housed at the Animal Center of Shanghai General Hospital. All animal protocols of this study were approved by the Research Animal Care and Use Committee of Shanghai General Hospital to Shanghai Jiao Tong University Medical School (Approval number: 2018KY201). Mice were anesthetized with 1% sodium pentobarbital (45 mg/kg) via intraperitoneal injection to ensure free of pain for any invasive operations.

Male C57BL/6 mice (7–8 weeks) were randomly divided into three groups (at least 5 mice per group): PBS + PBS group, PBS + LPS group and P12 + LPS group. Under anesthesia, the trachea was exposed via a median neck incision, and P12 (1 μM or 500 nM in 50 μL PBS) or the same volume of PBS was injected into the trachea through an insulin syringe (BD, USA) 2 h before LPS challenge (10 mg/kg, Sigma, USA) or PBS instillation. The wound was sutured, and the mice were recovered to normal activity until being sacrificed 24 h after LPS challenge to harvest bronchoalveolar lavage fluid (BALF), blood serum and lungs to examine cell infiltration, cytokine production and lung injury severity.

To deplete pulmonary macrophages, clodronate liposome (5 mg/mL, 75 μL/mouse) (Liposoma BV, the Netherlands) was given via intratracheal injection 72 h before P12 (500 nM, 50 μL/mouse) (or PBS) pre-treatment. Mice of the control group were administrated with the same volume of PBS liposome. After LPS challenge for 24 h, the same procedure described above was applied to assess the degree of inflammatory cell infiltration in ALI.

### BALF collection for differential leukocytes counting

Twenty-four hours after LPS challenge, bronchoalveolar lavage was performed by intratracheal injection of 0.8 mL cold sterile PBS through a 20-gauge catheter into the lungs, followed by solution withdrawing twice. The collected BALF was centrifuged at 1000 rpm at 4 ℃ for 10 min. The supernatant was stored at − 80 ℃ for further analysis, and the cell pellets were resuspended in PBS for total cell counting using a hemocytometer (cell suspensions were processed with the same volume of 3% glacial acetic acid to remove red blood cells). About 3 × 10^4^ BALF cells were spun down to a glass slide by cytospin (StatSpin, USA), and stained with Liu stain (Baso Diagnostics, Inc. Zhuhai, CHN) for differential leukocytes counting. More than 200 cells per slide were counted under a microscope. The remaining BALF cells were saved for flow cytometry analysis.

### Preparation of lung single cell suspensions

After BAL, lungs were dissected into pieces for enzyme digestion in RPMI-1640 medium containing DNase I (0.15 mg/mL, Sigma, USA) and collagenase II (1 mg/mL, Sigma, USA) at 37 ℃ for 1 h [27, 28]. The digested tissues were homogenized by vigorous shaking and passed through a 70-μm cell strainer (BD, USA). The cell suspensions were lysed with RBC Lysis Buffer (Beyotime, Shanghai, CHN) to remove red blood cells, and stained for flow cytometry analysis.

### Flow cytometry analysis

The BALF cells were stained with the flowing viability dye and fluorochrome-conjugated antibodies to examine the targeting ability of P12 to pulmonary macrophages: viability dye (L34962, Invitrogen, USA), CD3 (563024, BD), Gr1 (553126, BD), CD11c (562782, BD), F4/80 (123110, BioLegend), CD19 (557655, BD) and CD11b (101215, BioLegend). For investigating the effects of P12 on pulmonary macrophage polarization, the cells from the BALF and lung single cell suspensions were processed with FcBlock (562681, BD, USA) to reduce nonspecific binding and stained with the following: the viability dye and fluorochrome-conjugated antibodies against CD64 (139314, BioLegend, USA), CD11b (RM2817, Thermo Fisher Scientific, USA), CD11c (565872, BD), Siglec-F (562681, BD), MHC-II (107627, BioLegend), CD80 (104716, BioLegend) and CD206 (141712, BioLegend). Data were collected using LSRFortessa X30 (BD, USA) and analyzed with FlowJo software (TreeStar).

The BMDMs at day 7 were stained with anti-F4/80 (123110, BioLegend) and anti-CD11b (101215, BioLegend) antibodies to assess their purity after 7-day culture by looking at the percentage of CD11b^+^F4/80^+^ population using a BD Accuri C6 flow cytometer (BD Biosciences, USA).

### Histopathological analysis

In a separate experiment, the left lungs were dissected from mice without conducting BAL and fixed in 4% paraformaldehyde. The fixed lungs were embedded in paraffin and cut into 5 μm sections, which were stained with hematoxylin–eosin (H&E). These sections were imaged (at least six random fields) and blindly assessed by two independent researchers using the ALI scoring system described previously [29]. The indexes of lung injury were evaluated from the five histological features: (1) neutrophils in the alveolar space, (2) neutrophils in the interstitial space, (3) hyaline membranes, (4) proteinaceous debris filling the airspaces, and (5) alveolar septal thickening. Each was scored 0, 1, or 2 according to the injury severity. These five independent variables were weighted based on the relevance to ALI, and then were normalized to the number of fields. The final injury score is a continuous value between zero and one [29].

### Cytokine analysis

The cytokine levels of the BALF and serums were quantified using the Bio-plex Pro mouse cytokine 23-plex panel kit (M60009RDPD, Bio-Rad, USA). The measured cytokines were IL-1α, IL-1β, IL-2, IL-3, IL-4, IL-5, IL-6, IL-9, IL-10, IL-12p40, IL-12p70, IL-13, IL-17, Eotaxin, G-CSF, GM-CSF, IFN-γ, KC, MCP-1, MIP-1α, MIP-1β, RANTES and TNF-α. Multiplex assay was performed on the Bio-Plex 200 system (Bio-Rad, USA) according to manufacturer’s instructions.

In the in vitro BMDM experiments, the cytokines secreted by M1 macrophages (IL-12/23p40, IL-6 and IL-12p70) and M2 macrophages (IL-10) were quantified by ELISA (R&D Systems, USA) following the manufacturer’s protocols.

### Quantitative real-time PCR

Total RNA was extracted from BMDMs using TRIzol reagent (Invitrogen, USA) and converted to cDNA with PrimeScript™ RT reagent kit (Takara, Japan). The qRT-PCR was performed with FastStart Universal SYBR Green Master (Rox) (Roche, USA) in a QuantStudio™ 6 Flex Real-time System. Relative mRNA expression was quantitated with the 2^−△△Ct^ method. The primers designed for iNOS, IL-12, ARG1 and YM1 were listed in Additional file 1: Table S1; the primers of β-actin were obtained from Sangon Biotech (B661302, Shanghai, CHN).

### Statistical analysis

All the data were presented as means ± standard error of mean (SEM) from at least three independent experiments or biological replicates (N ≥ 3). Statistical analysis was performed using GraphPad Prism 7 by unpaired t-tests or one-way ANOVA with Bonferroni post hoc test when applicable. P value less than 0.05 was considered statistically significant.

## Results

### Fabrication and physicochemical characterization of peptide-coated GNPs

**Fig. 1:**
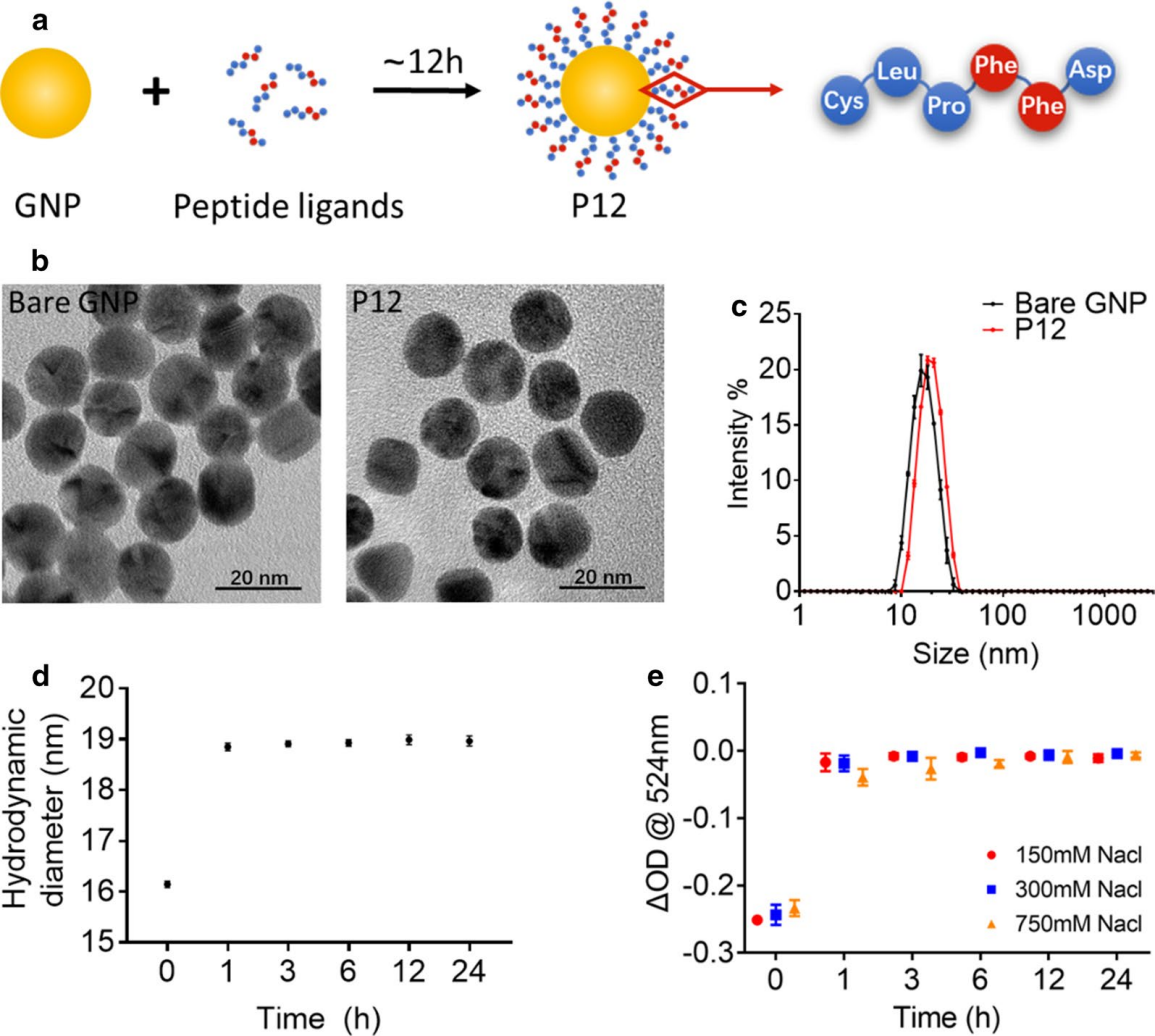
Fabrication of peptide-coated GNPs and their physicochemical characterization. **a** A schematic diagram of the synthesis of the peptide-coated GNP P12. **b** TEM images of bare GNPs and P12. **c** Size distribution of GNPs before and after peptide modification. **d** The change of hydrodynamic sizes of P12 as a function of incubation time after mixing the peptides with GNPs. **e** Stability of P12 formulated after various incubation time in different concentrations (150 to 750 mM) of NaCl solution. N = 3

In our previous study, we developed a novel anti-inflammatory peptide-GNP hybrid, P12, which was comprised of a 13 nm GNP core and a hexapeptide (amino acid sequence of CLPFFD) coating on the GNP surface (Fig. 1a). The peptide modification on the GNP surface was through the formation of the covalent-like bond between gold and the thiol group of the cysteine (C) residue at the N-terminal of the peptide sequence. Different from traditional nano-drug delivery system, P12 is a bioactive nanodevice that does not carry any therapeutic agent. However, the formation of peptide-coated GNP hybrids exhibits high potency in inhibiting TLR signaling and inflammatory reactions [21]. It was found that the two adjacent phenylalanine (FF) in the hexapeptide played an important role in determining the inhibitory activity of P12 from the structure–activity analysis by mutating “FF” to other amino acids.

The size and morphology of P12 were characterized by transmission electron microscopy (TEM) in comparison with bare GNPs. TEM images (Fig. 1b) showed that the GNPs were monodispersed with a uniform spherical structure and an average diameter of 13.0 ± 0.4 nm with or without peptide modification; this indicated that the hexapeptide coating did not change the appearance of GNPs. However, the hydrodynamic diameter of P12 was ~ 2.5 nm larger than the bare GNPs by DLS analysis (Fig. 1c), indicating the conjugation of peptide on the GNP surface. The zeta-potential of P12 (− 36.4 ± 0.3 mV) was slightly decreased compared with that of the bare GNPs (− 33.5 ± 0.8 mV) (Additional file 1: Figure S1). These negative charges (from the C-terminal aspartic acid (D) residues of the peptide) provided good colloidal stability of GNPs in aqueous solution.

To better understand the kinetics of peptide conjugation onto the GNPs, we measured the change of the hydrodynamic diameter of GNPs at different time points (up to 24 h) after mixing with the peptide solution. We found that the size of GNPs increased from 16.1 ± 0.1 to 18.8 ± 0.1 nm as short as 1 h after the addition of the peptide (Fig. 1d), suggesting that peptide conjugation onto GNP occurred within an hour. To ensure that such a fast conjugation process could form stable peptide-GNP hybrids, we further characterized their stability over time in NaCl solutions at various concentrations including 150 mM (physiological concentration as in the saline solution), 300 mM and 750 mM. As the GNPs become unstable and form aggregates, the optical absorption of GNPs at ~ 524 nm decreases. As shown in Fig. 1e, the unmodified GNPs (time 0) were not stable in any salt solution; with the presence of peptide in the solution for 1 to 6 h, the formed hybrid P12 was stable in 150 mM and 300 mM NaCl solution, but less stable in 750 mM NaCl solution. After a longer period of time (> 12 h), the hybrids became very stable even in 750 mM NaCl solution. These results suggested that the peptide could quickly coat on the GNP surface and stabilize GNPs as short as less than 1 h, but it took at least 12 h to maximize the peptide coatings and reach better stability in concentrated salt solution.

### The impacts of peptide-coated GNPs (P12) on global cytokine production in LPS-induced ALI mice

Our previous studies showed that P12 exhibited potent anti-inflammatory activity in vitro by inhibiting multiple TLR signaling pathways [21]. Its anti-inflammatory ability in vivo was also demonstrated in a LPS-induced ALI mouse model (Additional file 1: Figure S2). P12 was able to reduce total cell counts and neutrophil counts in the BALF, and decrease the severity of lung injury and inflammation by assessing pathological changes of lung tissues from five independent histological indexes: (1) neutrophils in the alveolar space, (2) neutrophils in the interstitial space, (3) formation of hyaline membranes, (4) proteinaceous debris filling the airspaces, and (5) alveolar septal thickening [29]. This in vivo anti-inflammatory action of P12 was also observed at lower P12 doses (500 and 250 nM) (Additional file 1: Figure S3).

**Fig. 2:**
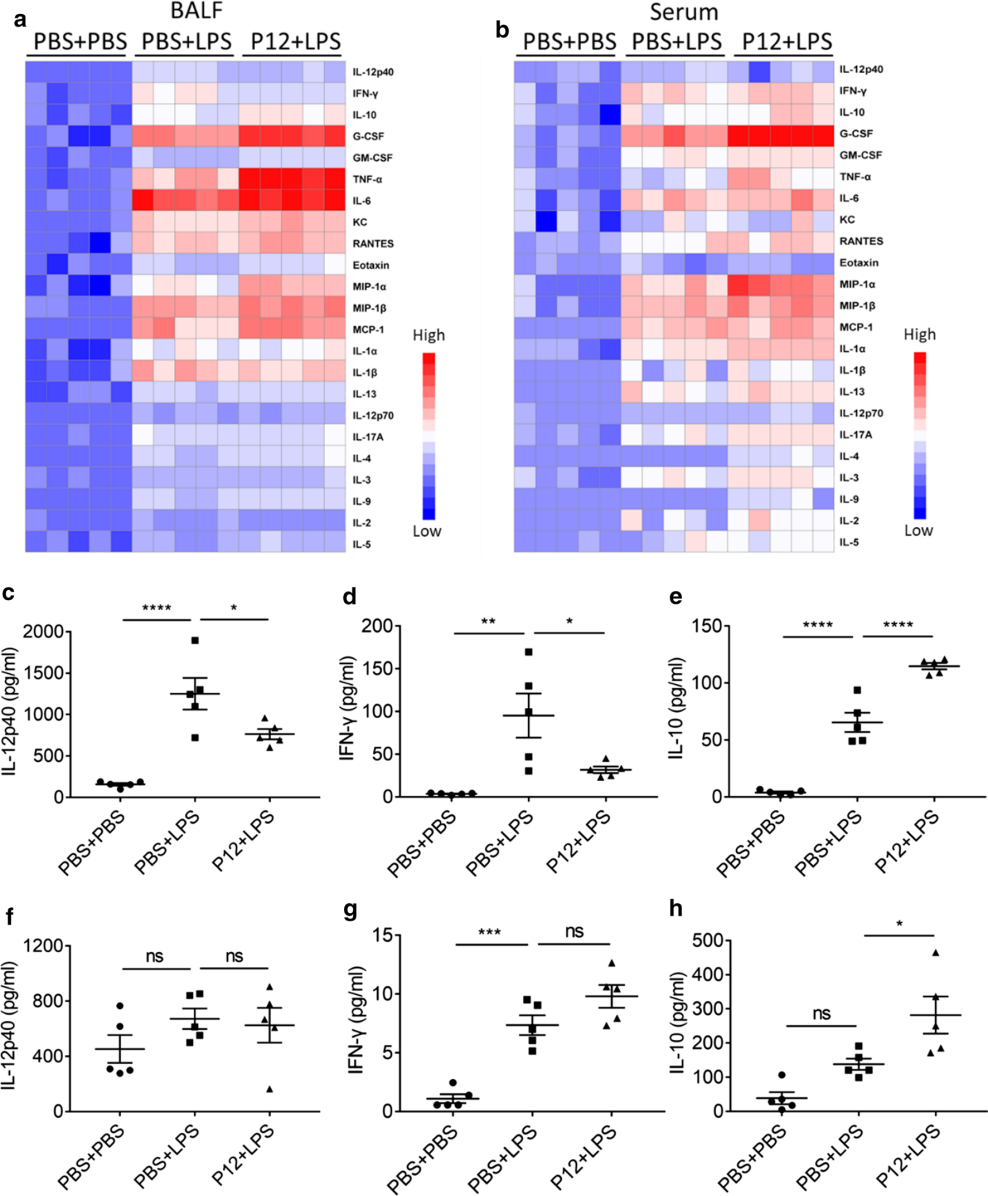
The effect of P12 on cytokine production in LPS-induced ALI mice. **a**, **b** The heat map showing cytokine profiles of the BALF (**a**) and serum (**b**) under LPS challenge (10 mg/kg, 24 h) with/without P12 pre-treatment (1 μM in 50 μL PBS, 2 h before LPS stimulation) using a multiplexed cytokine assay. The levels of selected cytokines of IL-12p40 (**c**, **f**), IFN-γ (**d**, **g**), and IL-10 (**e**, **h**) in the BALF (**c**–**e**) and in the serum (**f**–**h**) were shown. N = 5, ns: not significant, *p < 0.05, **p < 0.01, ***p < 0.001, ****p < 0.0001

Next, we employed multiplexed Luminex assay to evaluate the global impacts of P12 on the cytokine production in both BALF and serum of the ALI mice (Fig. 2). Compared with the LPS challenge group, P12 treatment could significantly decrease the levels of cytokines associated with M1 phenotype such as IL-12p40 and IFN-γ (Fig. 2a, c, d), while increasing those related to M2 phenotype such as IL-10 (Fig. 2a, e) in the BALF. Such effects were not significant in serum (Fig. 2b, f, g) except the increase of IL-10 (Fig. 2h). Of note, the level of granulocyte-colony stimulating factor (G-CSF) that has been reported to have an anti-inflammatory role was also significantly elevated by P12 (Fig. 2a, b and Additional file 1: Figure S4) in the BALF and serum [30–33]. Interestingly, the M2 inducing cytokines IL-4 and IL-13 were significantly elevated in the serum (Additional file 1: Figure S4). These results suggested that in addition to its TLR inhibitory activity, the anti-inflammatory effects of P12 treatment may also involve macrophage polarization from inflammatory M1 phenotype toward anti-inflammatory M2 phenotype.

### P12 targeted pulmonary macrophages to reduce inflammation

**Fig. 3:**
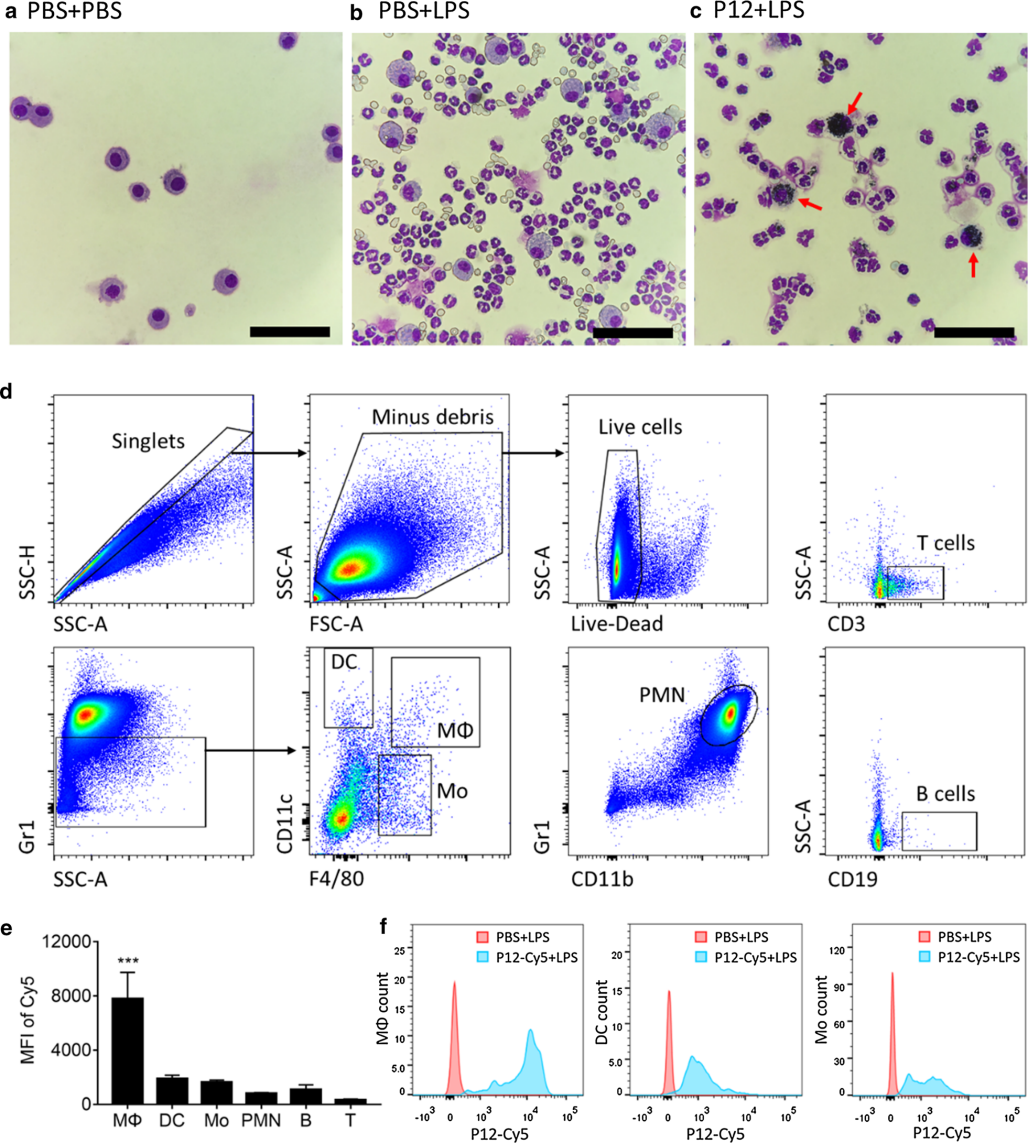
The preferential uptake of P12 by pulmonary macrophages upon intratracheal administration. Liu staining of cells in the BALF collected from three experimental groups: **a** PBS + PBS, **b** PBS + LPS, and **c** P12 + LPS group; the red arrows indicate pulmonary macrophages with internalized P12 in dark blue color; scale bar represents 60 µm. **d** Gating strategy of flow cytometry analysis to identify monocytes (Mo), dendritic cells (DCs), macrophages (Mφ), neutrophils, T cells and B cells in the BALF. **e** The amount of internalized P12 in various immune cells in the BALF using the mean fluorescence intensity (MFI) of Cy5-labeled P12 (P12-Cy5); N = 4, ***p < 0.001. **f** Uptake of P12-Cy5 nanoparticles in mononuclear phagocytic immune cells: macrophages (left), DCs (middle) and monocytes (right); the P12-Cy5 treated group shown in blue and the corresponding untreated group shown in red

In order to verify that P12 primarily targeted pulmonary macrophages to reduce inflammation of ALI, we first analyzed preferential uptake of P12 in different cell types in the BALF. It was clearly seen from the cytospin BAL cells that P12 was mainly accumulated in the macrophage-like cells as indicated by the red arrows (Fig. 3a–c). Through labelling P12 with trace of a fluorescent probe PEG-Cy5 (P12-Cy5) [22] and staining the BAL cells with markers to distinguish different immune cell types (Fig. 3d), we were able to apply flow cytometry analysis to quantify the preferential uptake of P12 by the following cell types: T cells (CD3^+^), B cells (CD19^+^), neutrophils (Gr1^high^CD11b^+^), dendritic cells (F4/80^−^CD11c^high^Gr1^low^), macrophages (F4/80^+^CD11c^+^Gr1^−^) and monocytes (F4/80^low^CD11c^−^Gr1^low^) [34, 35]. By comparing the mean fluorescence intensity (MFI) of Cy5 among these cell types, we found that BAL macrophages had the highest MFI (Fig. 3e, f), indicating that P12 primarily targeted pulmonary macrophages after intratracheal administration.

**Fig. 4:**
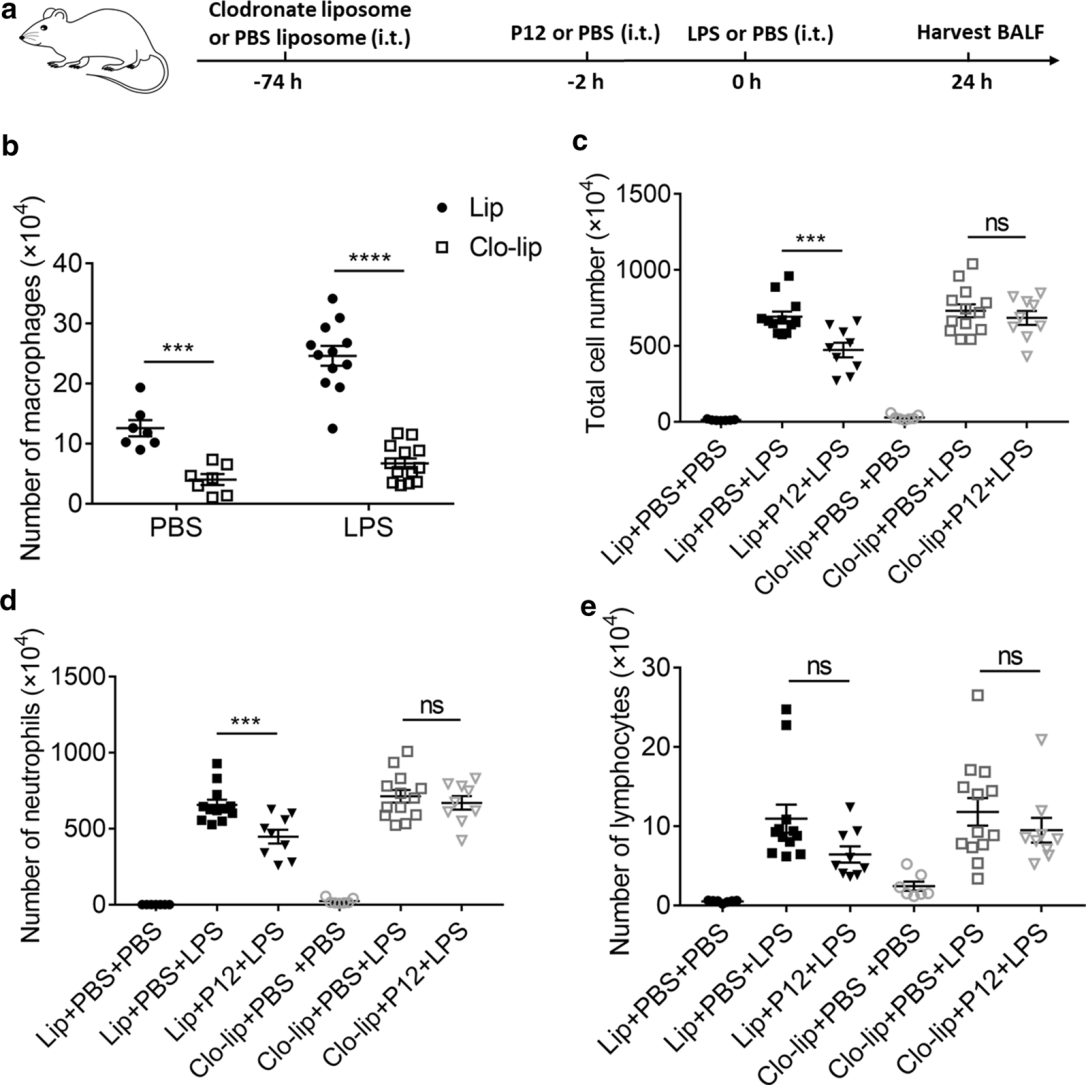
The importance of macrophages in the anti-inflammatory effects of P12 in the LPS-induced ALI mouse model. **a** The experimental procedure of macrophage depletion by clodronate liposomes (75 μL, 5 mg/mL) prior to P12 pre-treatment (50 μL, 500 nM) and LPS challenge. PBS was given as a control. At 24 h after LPS stimulation, the BALF was harvested to assess the efficiency of macrophage depletion (**b**), and to analyze the number of total cells (**c**), neutrophils (**d**), and lymphocytes (**e**) in each group. N ≥ 7, Lip: PBS encapsulating liposomes, Clo-lip: clodronate encapsulating liposomes, ns: not significant, ***p < 0.001, ****p < 0.0001

To confirm whether the anti-inflammatory action of P12 was mainly through pulmonary macrophages, they were removed by intratracheal injection of a well-known macrophage depletion agent, clodronate liposome [36, 37]. Clodronate liposome was administered 72 h before P12 pre-treatment to ensure an effective removal of pulmonary macrophages, and the mice were challenged with LPS 2 h after P12 treatment (Fig. 4a). Blank liposomes (i.e., PBS liposomes) were given at the same volume as the vehicle control. At 24 h after LPS stimulation, the BALF was collected to assess the depletion of macrophages and the cell infiltration. As seen in Fig. 4b, the intratracheal administration of clodronate liposomes led to about 70% reduction in BAL macrophages in both control group (PBS group) and LPS group, confirming the effective removal of lung macrophages. In the case of macrophage depletion, P12 treatment could not reduce the total cell counts (Fig. 4c) and neutrophil counts (Fig. 4d) induced by LPS challenge, whereas the protective effect of P12 still remained in the presence of lung macrophages (blank liposome pre-treatment). Note that P12 intervention had no effect on the number of BAL lymphocytes with or without macrophage depletion (Fig. 4e). These results demonstrated the importance of pulmonary macrophages in P12-mediated attenuation of inflammatory cell infiltration in ALI.

### Effects of P12 on the polarization of BMDMs

It is known that the activation of pulmonary macrophages played a critical role in the initiation and development of ALI/ARDS [8]. Recent studies suggested that reprograming macrophages toward M2 phenotype may be an effective strategy to treat ALI/ARDS and other inflammatory diseases [38, 39]. Our previous study showed that P12 intervention increased the number of regulatory T (Treg) cells in the lung and draining lymph nodes of ALI mice [24]. Growing evidences suggested a positive correlation between Treg cells and M2 macrophages [40, 41]. As P12 primarily targeted pulmonary macrophages to reduce lung inflammation (Figs. 3 and 4) and reshaped the cytokine production profile toward the anti-inflammation phase (Fig. 2), we hypothesized that P12 treatment could induce M2 macrophages in addition to its TLR inhibitory activity to reduce inflammatory responses.

**Fig. 5:**
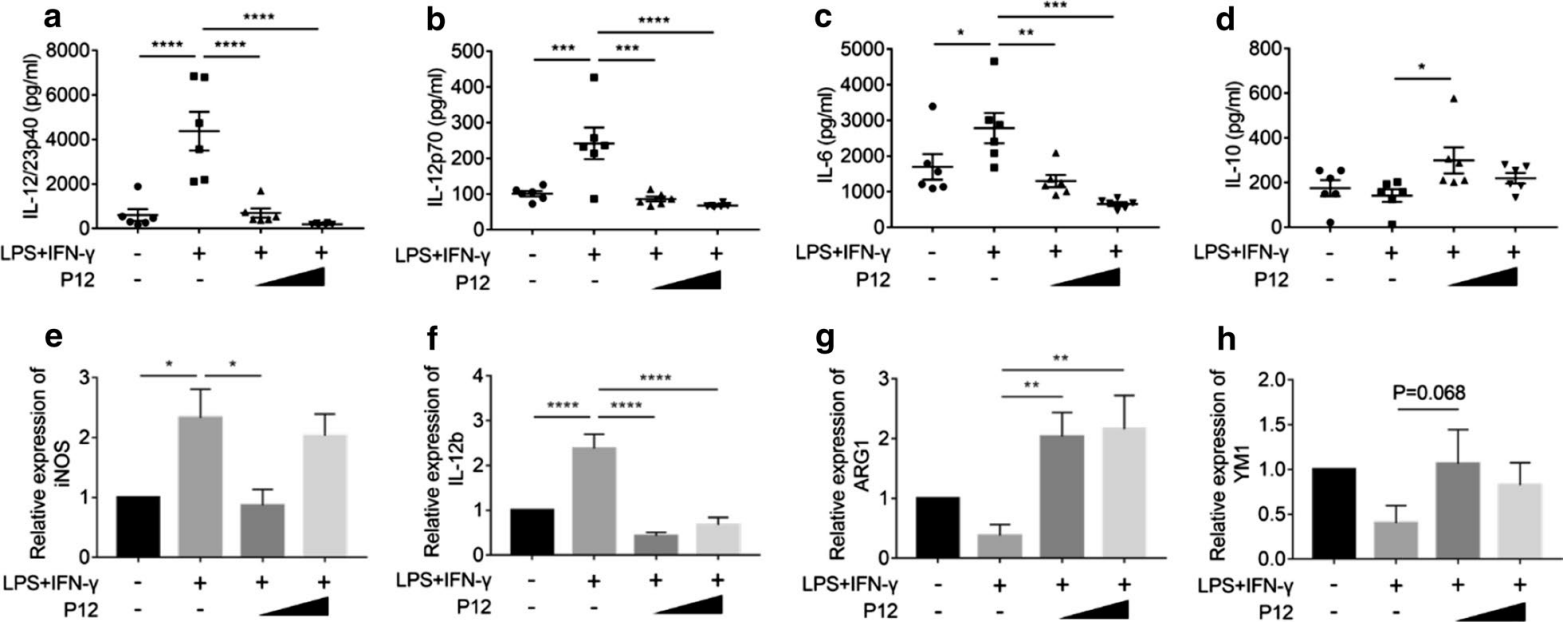
The effects of P12 on the polarization of BMDMs to M1 phenotype. Cytokine levels of IL-12/23p40 (**a**), IL-12p70 (**b**), IL-6 (**c**) and IL-10 (**d**) were quantified by ELISA in the macrophage culture supernatants collected 24 h after LPS and IFN-γ co-stimulation for M1 polarization with/without P12 treatment. The mRNA levels of M1 markers of iNOS (**e**) and IL-12 (**f**), and the M2 markers of ARG1 (**g**) and YM1 (**h**) were analyzed by qRT-PCR. P12 concentration = 12.5 and 50 nM, N = 6, *p < 0.05, **p < 0.01, ***p < 0.001, ****p < 0.0001

To test this hypothesis, we cultured mouse BMDMs and examined the effects of P12 treatment on the macrophage polarization ex vivo. Mouse bone marrow cells were collected and derived (using M-CSF) into macrophages (BMDMs) after 7-day culture (Additional file 1: Figure S5a), with a purity reaching above 90% (Additional file 1: Figure S5b) prior to further polarization experiments. In response to different stimuli, BMDMs can be polarized into distinct functional phenotypes including the pro-inflammatory (M1) or the anti-inflammatory (M2) phenotype. BMDMs were first stimulated with LPS and IFN-γ to become M1 macrophages in the presence and absence of two different concentrations (12.5 and 50 nM) of P12 for 24 h. In the absence of P12, LPS and IFN-γ stimulation significantly increased the pro-inflammatory cytokines IL-12/23p40, IL-12p70 and IL-6 production, which were mainly secreted by M1 macrophages. At both concentrations, P12 was able to reduce the levels of these pro-inflammatory cytokines (Fig. 5a–c). Interestingly, P12 at low concentration was also able to promote the anti-inflammatory cytokine IL-10 secretion, while the co-stimulation alone by LPS and IFN-γ had no effect (Fig. 5d). At gene expression level, the expression of M1 markers iNOS and IL-12 elevated by the co-stimulation was significantly decreased upon P12 treatment (Fig. 5e and f); on the other hand, the M2 marker ARG1 expression was significantly increased by P12 (Fig. 5g), and YM1 level was elevated but not statistically significant (Fig. 5h). It is worth noting that P12 treatment did not affect the viability of BMDMs during M1 or M2 polarization (Additional file 1: Figure S6). These results suggested that P12 treatment could reduce the pro-inflammatory cytokines secretion and M1 specific makers expression but promote the anti-inflammatory cytokine production and elevate the M2 specific marker expression during the M1 polarization process.

**Fig. 6:**
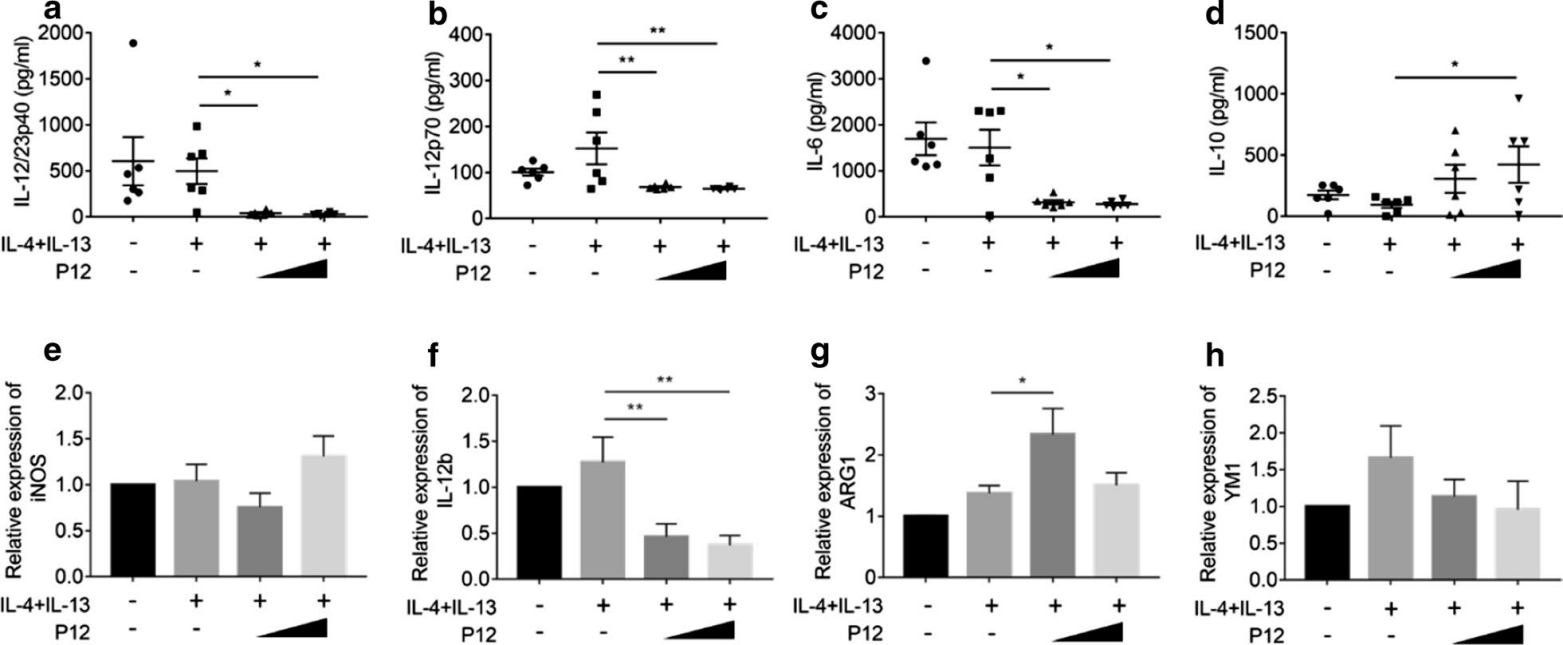
The effects of P12 on the polarization of BMDMs to M2 phenotype. Under IL-4 and IL-13 co-stimulation for M2 polarization, cytokine levels of IL-12/23p40 (**a**), IL-12p70 (**b**), IL-6 (**c**) and IL-10 (**d**) in the macrophage culture supernatants were quantified by ELISA 24 h after stimulation in the presence or absence of P12 treatment. The mRNA levels of M1 markers of iNOS (**e**) and IL-12 (**f**), and the M2 markers of ARG1 (**g**) and YM1 (**h**) were analyzed by qRT-PCR. P12 concentration = 12.5 and 50 nM, N = 6, *p < 0.05, **p < 0.01

Next, we evaluated the effects of P12 treatment on the M2 polarization under IL-4 and IL-13 co-stimulation. We found that IL-4 and IL-13 co-stimulation did not alter the levels of IL-12/23p40, IL-12p70 and IL-6 as expected; however, the P12 treatment (at both low and high concentrations) could significantly reduce the baseline of these pro-inflammatory cytokines (Fig. 6a–c). The high concentration of P12 could significantly increase the IL-10 level (Fig. 6d). By analyzing the characteristic gene expression of M1 and M2 macrophages, we found that P12 did not affect iNOS gene expression (Fig. 6e) during M2 polarization, but was able to reduce the baseline of M1 characteristic gene expression of IL-12 (Fig. 6f) while increasing the M2 characteristic gene expression of ARG1 (Fig. 6g). The expression of another M2 polarization marker YM1 was not affected by P12 during M2 polarization process. It should be noted that the co-stimulation of IL-4 and IL-13 did not increase the IL-10 production as well as the expression of M2 gene markers simply because the derived BMDMs by M-CSF prior to any co-stimulation had intrinsic characteristics close to M2 phenotype [42, 43]. These results demonstrated that P12 treatment could further reduce the baseline of pro-inflammatory cytokines secretion and M1 specific gene expressions, and at the same time enhance the anti-inflammatory cytokine production and the M2 specific gene expression under M2 polarization process induced by IL-4 and IL-13 co-stimulation. Altogether, these results provided strong evidence that P12 was capable of driving the polarization of BMDMs from M1 toward M2 phenotype ex vivo.

### P12 enhanced M2 pulmonary macrophage polarization during LPS-induced ALI

**Fig. 7:**
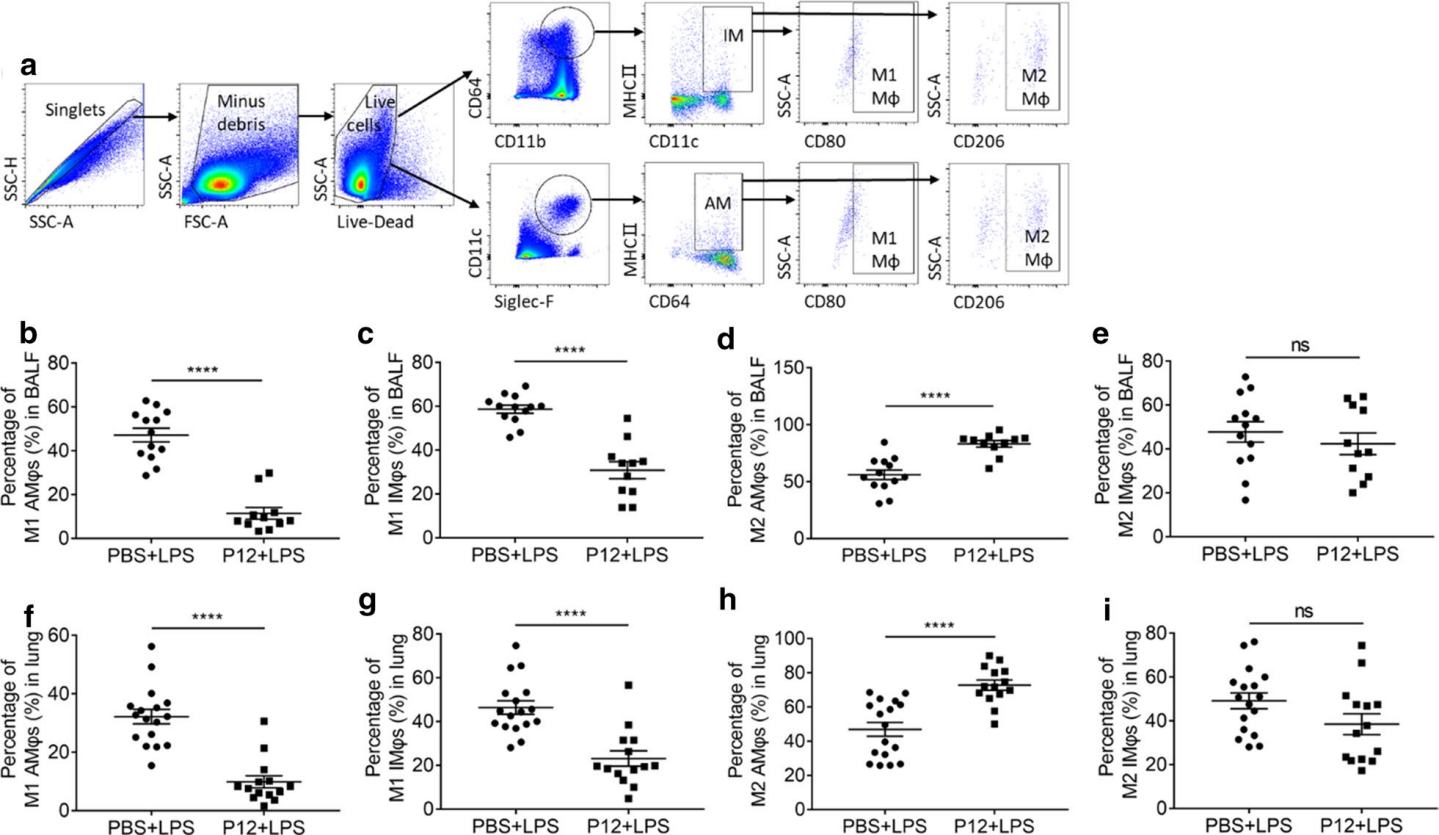
The effects of P12 on pulmonary macrophage polarization in the LPS-induced ALI mouse model. **a** Gating strategy of flow cytometry analysis to identify the M1 and M2 phenotypes of interstitial macrophages (IMs) and tissue-resident alveolar macrophages (AMs) in the BALF and single cell suspensions of lung tissues. Percentages of M1 macrophages in AM (**b**, **f**) and IM (**c**, **g**) populations, and M2 macrophages in AM (**d**, **h**) and IM (**e**, **i**) in the BALF (**b**–**e**) as well as in the lung (**f**–**i**) upon P12 treatment compared to the LPS group. N ≥ 11, ns: not significant, ****p < 0.0001

The M2 macrophage polarization ability of P12 was further confirmed in vivo in a LPS-induced ALI mouse model. The phenotypes of the tissue resident alveolar macrophages (AMs) and the alveolar septum (or alveolar corner) interstitial space located interstitial macrophages (IMs) were analyzed by flow cytometry on their specific markers (Fig. 7a): Siglec-F^+^CD11c^+^CD64^+^MHCII was used to define AMs, and IMs were referred to as CD11b^+^CD11c^+^CD64^+^MHCII^+^ population; in addition, the CD80^+^ or CD206^+^ subset in the AM and IM populations was considered as M1 or M2 macrophages, respectively. The analysis of BAL cells revealed that P12 pre-treatment significantly reduced the percentage of M1 macrophages in both AM (Fig. 7b) and IM (Fig. 7c) populations; the percentage of M2 macrophages in AMs (Fig. 7d) was increased but that in IMs was not (Fig. 7e). These phenomena were also seen in the single cell suspensions collected from the lung tissues (Fig. 7f–i). It should be noted that because the control mice (PBS + PBS group) were not challenged by LPS to induce inflammation, infiltrated cells in both the BALF and lung tissues of the control mice were too few for the multi-color flow analysis to define the polarization phenotypes of AMs and IMs.

Through immunofluorescence staining on the lung tissue sections (Additional file 1: Figure S7), it was seen that the CD206^+^ macrophages (M2 type) were relatively abundant in the control mice (PBS + PBS group), but diminished upon LPS stimulation, indicating M1 macrophage polarization. With P12 treatment, more CD206^+^ macrophages were observed, indicating M2 macrophage polarization (Additional file 1: Figure S7a). In addition, compared with the control group, the iNOS^+^ macrophages were increased by LPS stimulation but reduced by P12 treatment (Additional file 1: Figure S7b). Collectively, these results confirmed that P12 treatment was able to promote the polarization of pulmonary macrophages to M2 phenotype, contributing to controlling the inflammatory responses in ALI.

## Discussion

ALI/ARDS is commonly seen in the ICU clinics, characterized with overwhelming uncontrolled inflammatory reactions in the lung. However, over 50 years have passed since it was first described, there is still no effective pharmacological therapies for ALI/ARDS in clinics [7]. As more and more evidences demonstrated that pulmonary macrophages play a critical role in the initiation, development and resolution of inflammation in ALI/ARDS, regulation of macrophage activation and polarization in the lung would provide a promising therapeutic strategy to manage such detrimental conditions. In this study, we showed that a special class of peptide-coated GNPs could reduce inflammatory responses of BMDMs and promote their polarization from inflammatory M1 toward anti-inflammatory M2 phenotype. In the ALI mouse model, we discovered that the intratracheal administration of the nanoparticles significantly alleviated lung injuries by targeting pulmonary macrophages to reduce their inflammatory reactions and polarize them toward an anti-inflammatory/reparative M2 phenotype. The M2 macrophage polarization capability together with the intrinsic TLR inhibitory activity make these peptide-coated GNPs potential next generation potent anti-inflammatory therapeutics for ALI/ARDS.

### Importance of targeting pulmonary macrophage for ALI/ARDS

Pulmonary macrophages are mononuclear phagocytic innate immune cells, which reside in the alveoli and lung tissue interstitium, named alveolar macrophages (AMs) and interstitial macrophages (IMs), respectively [9]. Similar to other macrophages in general, pulmonary macrophages are functionally plastic and can rapidly adopt different phenotypes based on the stimuli they received in their surroundings. For instance, they can differentiate toward an inflammatory M1 phenotype secreting various pro-inflammatory cytokines (e.g., IL-6, IL-12 and TNF-α). This is essential for the defense against invading pathogens, but also contributes to tissue destruction. On another extreme, they can polarize to become an immunosuppressive M2 phenotype, which produces high level of anti-inflammatory cytokine IL-10 and mediates tissue repair. Their unique plasticity and the balance in between different phenotypes maintain the immune homeostasis in the lung under healthy conditions.

In ALI/ARDS, AMs are involved in the pathogenesis of the disease development over time [44]. The development of ALI/ARDS generally consists of three phases: exudative phase (within 24 h) of initial injury responses, proliferative phase for tissue repairing, and the final fibrotic phase [8]. In the exudative phase, AMs are activated by recognition of microbial molecular patterns or danger signals from tissue injury and differentiated into M1 phenotype that drives acute inflammatory responses. They are capable of releasing inflammatory cytokines, recruiting neutrophils and monocytes/macrophages to the lungs, and interacting with other cells (e.g., lymphocytes, epithelial and endothelial cells) to enhance the inflammatory responses [44, 45]. This eventually leads to tissue injury and pulmonary edema. In the later phases, AMs can switch into an anti-inflammatory M2 phenotype, which assists inflammation resolution and tissue repair [45]. Therefore, AMs remain as an ideal cellular target for pharmacologic intervention of ALI/ARDS.

Nanodevices are thought to be ideal agents targeting macrophages owing to their intrinsic nanoscale property suitable for phagocytosis [18, 19]. In this study, we presented a bioactive peptide-coated GNP—P12 that was primarily taken up by macrophages in the lung (Fig. 3). P12 was able to potently inhibit macrophage activation through attenuating TLR signaling cascades [21] and reduce LPS-induced pulmonary inflammation (Additional file 1: Figure S2) [24]. When the pulmonary macrophages were depleted, the anti-inflammatory effect of P12 was abolished (Fig. 4), indicating that pulmonary macrophages indeed were indispensable for regulating the inflammatory responses by our newly developed peptide-GNP hybrids in an ALI mouse model. More importantly, P12 was capable of promoting macrophage polarization toward an anti-inflammatory and pro-resolution M2 phenotype that facilitated the control of pulmonary inflammation during ALI (Figs. 5, 6, 7). The induction of AM polarization to a M2 phenotype has been employed in other studies, where administration of IL-4 could reprogram the endogenous inflammatory macrophages to anti-inflammatory ones, and accelerate inflammation resolution and lung repair in a STAT6-dependent manner in both LPS and *Pseudomonas aeruginosa* bacterial pathogen-induced ALI mouse models [13]. This suggests that targeting pulmonary macrophages could be an effective strategy to manage ALI and maintain the immune homeostasis in the lung.

### Possible mechanism(s) of M2 macrophage polarization by peptide-coated GNPs

This study discovered M2 macrophage polarization as a new acting mechanism of P12 in controlling inflammatory responses during ALI. Previously, we have shown that P12 can potently inhibit TLR4 signaling mediated NF-κB and IRF3 activation [21]. It has been reported that NF-κB inhibition in macrophages could lead to the polarization toward M2 phenotype [46], which supports our observation in current study. In addition, from our previous results of the transcriptomic analysis of P12 in human PBMC, an important transcription factor of M1 polarization, STAT1, was down regulated by P12 [22], providing evidence of P12 regulating macrophage polarization preferentially into M2 phenotype at the transcription level.

Macrophage polarization can also be regulated by the surrounding stimuli, particularly cytokines, in the microenvironment. We found that P12 treatment resulted in significant enhanced G-CSF production in the lung (Additional file 1: Figure S4). The cytokine G-CSF was reported to exhibit anti-inflammatory activity and enhance alveolar epithelial wound repair as well as facilitate clearance of apoptotic cells by macrophages in animal models [47]. Interestingly, it was found that low concentration of G-CSF could down-regulate the expression of the M1 driving cytokine IFN-γ, while high concentration of G-CSF could up-regulate the production of M2 driving cytokine IL-4 in activated T cells [32]. Indeed, the elevated IL-4 levels were observed in mouse serum (Additional file 1: Figure S4). Thus, elevated G-CSF production by P12 may have dual effects on both promoting macrophage polarization into M2 phenotype and reducing the excessive and damaging inflammatory responses in the lung. In addition, we found that P12 treatment led to significantly enhanced IL-13 production in the serum, which may also contribute to the induction of M2 macrophage.

It is worth noting that the M1 related cytokines TNF-α and IL-1β were not reduced by P12 in both BMDMs studies and the ALI models. One possible explanation was that the manipulation of macrophage activation by P12 was mainly through TLR4 inhibition. TLR4 signaling cascade has two arms of pathways: MyD88-dependent and TRIF-dependent pathways; the former primarily drives NF-κB activation while the latter activates IRF3. Since the peptide-coated GNPs were mainly taken up into the endosomal compartments and blocked their acidification [21], we speculated that P12 primarily inhibited the TRIF-dependent pathway that relies on signals from the endosomes, and hence not affected the production of TNF-α and IL-1β, which is predominantly controlled by MyD88-dependent NF-κB activation [48, 49].

Taken together, our findings suggest that P12 acts through two main strategies on lung macrophages to intervene inflammatory responses during the induction and resolution phases of ALI/ARDS. One is to prevent macrophage over activation in the early phase of ALI/ARDS by inhibiting TLR signaling. The other is to reprogram the inflammatory M1 macrophages into the anti-inflammatory and pro-resolution/reparative M2 phenotypes. However, it remains unclear whether the P12 enabled polarization of M2 macrophages is persistent or transient; therefore, understanding how long the protective role of P12 can last and what the determining factors might be would help design better peptide-coated GNPs for clinical translation as our future endeavor.

### Advantages of using nanodevices in manipulating macrophage polarization for ALI/ARDS

Nanodevices provide several advantages to manipulate macrophage polarization in the lung. First, nanodevices can be directly administered into lungs by inhalation or instillation as a local therapy; thus, they can bypass the first-pass metabolism effect of hepatobiliary clearance. Second, nanodevices are capable of targeting macrophages by nature due to their innate phagocytotic activity of nano-sized particles. On top of these two, our designed peptide-coated GNPs have additional advantages: they can be quickly formulated (within 1 h) and have a good stability profile in the physiological condition (Fig. 1); they utilize multiple mechanisms of action to regulate lung inflammation, protect lung from injuries and promote inflammation resolution. These overall properties make such nanodevices a new generation of powerful therapeutics to treat ALI.

Although the nanotechnology-based systems described herein hold promise for the future design of nanotherapeutic interventions for ALI/ARDS, it should be noted that macrophage-targeted therapies have some intrinsic limitations. For example, the long-term effectiveness of such method is highly dependent on the stability/plasticity of the polarized macrophages, which affects its therapeutic window. Therefore, nanodevices that allow long-term repeated administration may be ideal for continuous macrophage polarization in the lung in ALI/ARDS.

## Conclusions

The activation and polarization of macrophages play an important role in the initiation and resolution of inflammation, respectively, in ALI/ARDS. These studies showed a bioactive nanodevice that is made of hexapeptides and GNPs forming peptide-coated GNP hybrids with capability of targeting lung macrophages upon intratracheal administration to the airway. Most importantly, they were able to reduce macrophage activation and promote M1-to-M2 phenotype transition in the cultured BMDMs, as well as in the lung of ALI mice. Our results indicated that the designed peptide-coated GNPs adopt multiple mechanisms of actions to regulate lung inflammation, protect lung from injuries and promote inflammation resolution, making such therapeutic nanodevices a new generation of powerful therapeutics to treat ALI/ARDS.

## Supplementary information

**Additional file 1.** Additional figures describe the zeta-potential of P12, the anti-inflammatory activities of P12 in LPS-induced ALI mice at different doses, the culture procedure and purity of BMDMs, viability of M1 and M2 macrophages by P12 treatment, and the changes of the M2 and M1 macrophage percentage in the lung tissues upon P12 treatment by immunofluorescence imaging.

## Data Availability

All data generated and analyzed during this research are included in this article.

## References

[1] Bellani G, Laffey JG, Pham T, Fan E, Brochard L, Esteban A, Gattinoni L, van Haren F, Larsson A, McAuley DF (2016). Epidemiology, patterns of care, and mortality for patients with acute respiratory distress syndrome in intensive care units in 50 countries. JAMA.

[2] Riviello ED, Kiviri W, Twagirumugabe T, Mueller A, Banner-Goodspeed VM, Officer L, Novack V, Mutumwinka M, Talmor DS, Fowler RA (2016). Hospital incidence and outcomes of the acute respiratory distress syndrome using the Kigali modification of the Berlin definition. Am J Respir Crit Care Med.

[3] Fan E, Brodie D, Slutsky AS (2018). Acute respiratory distress syndrome: advances in diagnosis and treatment. JAMA.

[4] Mac Sweeney R, McAuley DF (2016). Acute respiratory distress syndrome. Lancet.

[5] Brower RG, Matthay MA, Morris A, Schoenfeld D, Thompson BT, Wheeler A (2000). Ventilation with lower tidal volumes as compared with traditional tidal volumes for acute lung injury and the acute respiratory distress syndrome. N Engl J Med.

[6] Villar J, Kacmarek RM, Perez-Mendez L, Aguirre-Jaime A (2006). A high positive end-expiratory pressure, low tidal volume ventilatory strategy improves outcome in persistent acute respiratory distress syndrome: a randomized, controlled trial. Crit Care Med.

[7] Shaw TD, McAuley DF, O'Kane CM (2019). Emerging drugs for treating the acute respiratory distress syndrome. Expert Opin Emerg Drugs.

[8] Thompson BT, Chambers RC, Liu KD (2017). Acute respiratory distress syndrome. N Engl J Med.

[9] Huang X, Xiu H, Zhang S, Zhang G (2018). The role of macrophages in the pathogenesis of ALI/ARDS. Mediat Inflamm.

[10] Song C, Li H, Li Y, Dai M, Zhang L, Liu S, Tan H, Deng P, Liu J, Mao Z (2019). NETs promote ALI/ARDS inflammation by regulating alveolar macrophage polarization. Exp Cell Res.

[11] Lu HL, Huang XY, Luo YF, Tan WP, Chen PF, Guo YB (2018). Activation of M1 macrophages plays a critical role in the initiation of acute lung injury. Biosci Rep..

[12] Arora S, Dev K, Agarwal B, Das P, Syed MA (2018). Macrophages: their role, activation and polarization in pulmonary diseases. Immunobiology.

[13] D'Alessio FR, Craig JM, Singer BD, Files DC, Mock JR, Garibaldi BT, Fallica J, Tripathi A, Mandke P, Gans JH (2016). Enhanced resolution of experimental ARDS through IL-4-mediated lung macrophage reprogramming. Am J Physiol Lung Cell Mol Physiol.

[14] Ying W, Cheruku PS, Bazer FW, Safe SH, Zhou B (2013). Investigation of macrophage polarization using bone marrow derived macrophages. J Vis Exp.

[15] Murray PJ (2017). Macrophage polarization. Annu Rev Physiol.

[16] Bejerano T, Etzion S, Elyagon S, Etzion Y, Cohen S (2018). Nanoparticle Delivery of miRNA-21 mimic to cardiac macrophages improves myocardial remodeling after myocardial infarction. Nano Lett.

[17] Kim B, Pang HB, Kang J, Park JH, Ruoslahti E, Sailor MJ (2018). Immunogene therapy with fusogenic nanoparticles modulates macrophage response to *Staphylococcus aureus*. Nat Commun.

[18] Parayath NN, Parikh A, Amiji MM (2018). Repolarization of tumor-associated macrophages in a genetically engineered nonsmall cell lung cancer model by intraperitoneal administration of hyaluronic acid-based nanoparticles encapsulating microrna-125b. Nano Lett.

[19] Mehanna MM, Mohyeldin SM, Elgindy NA (2014). Respirable nanocarriers as a promising strategy for antitubercular drug delivery. J Control Release.

[20] Libutti SK, Paciotti GF, Byrnes AA, Alexander HR, Gannon WE, Walker M, Seidel GD, Yuldasheva N, Tamarkin L (2010). Phase I and pharmacokinetic studies of CYT-6091, a novel PEGylated colloidal gold-rhTNF nanomedicine. Clin Cancer Res.

[21] Yang H, Fung SY, Xu S, Sutherland DP, Kollmann TR, Liu M, Turvey SE (2015). Amino acid-dependent attenuation of toll-like receptor signaling by peptide-gold nanoparticle hybrids. ACS Nano.

[22] Yang H, Kozicky L, Saferali A, Fung SY, Afacan N, Cai B, Falsafi R, Gill E, Liu M, Kollmann TR (2016). Endosomal pH modulation by peptide-gold nanoparticle hybrids enables potent anti-inflammatory activity in phagocytic immune cells. Biomaterials.

[23] Yang H, Fung SY, Bao A, Li Q, Turvey SE (2017). Screening bioactive nanoparticles in phagocytic immune cells for inhibitors of toll-like receptor signaling. J Vis Exp.

[24] Xiong Y, Gao W, Xia F, Sun Y, Sun L, Wang L, Ben S, Turvey SE, Yang H, Li Q (2018). Peptide-gold nanoparticle hybrids as promising anti-inflammatory nanotherapeutics for acute lung injury: in vivo efficacy, biodistribution, and clearance. Adv Healthc Mater.

[25] Storhoff JJ, Elghanian R, Mucic RC, Mirkin CA, Letsinger RL (1998). One-Pot Colorimetric differentiation of polynucleotides with single base imperfections using gold nanoparticle probes. J Am Chem Soc.

[26] Kozicky L, Sly LM (2017). Assessment of antibody-based drugs effects on murine bone marrow and peritoneal macrophage activation. J Vis Exp.

[27] Cai T, Qiu J, Ji Y, Li W, Ding Z, Suo C, Chang J, Wang J, He R, Qian Y (2019). IL-17-producing ST2(+) group 2 innate lymphoid cells play a pathogenic role in lung inflammation. J Allergy Clin Immunol.

[28] Shibata S, Miyake K, Tateishi T, Yoshikawa S, Yamanishi Y, Miyazaki Y, Inase N, Karasuyama H (2018). Basophils trigger emphysema development in a murine model of COPD through IL-4-mediated generation of MMP-12-producing macrophages. Proc Natl Acad Sci U S A.

[29] Matute-Bello G, Downey G, Moore BB, Groshong SD, Matthay MA, Slutsky AS, Kuebler WM (2011). An official American Thoracic Society workshop report: features and measurements of experimental acute lung injury in animals. Am J Respir Cell Mol Biol.

[30] Tanaka H, Nishino M, Nakamori Y, Ogura H, Ishikawa K, Shimazu T, Sugimoto H (2001). Granulocyte colony-stimulating factor (G-CSF) stiffens leukocytes but attenuates inflammatory response without lung injury in septic patients. J Trauma.

[31] Jian MY, Koizumi T, Tsushima K, Fujimoto K, Kubo K (2004). Effects of granulocyte colony-stimulating factor (G-CSF) and neutrophil elastase inhibitor (ONO-5046) on acid-induced lung injury in rats. Inflammation.

[32] Malashchenko VV, Meniailo ME, Shmarov VA, Gazatova ND, Melashchenko OB, Goncharov AG, Seledtsova GV, Seledtsov VI (2018). Direct anti-inflammatory effects of granulocyte colony-stimulating factor (G-CSF) on activation and functional properties of human T cell subpopulations in vitro. Cell Immunol.

[33] Hartung T (1998). Anti-inflammatory effects of granulocyte colony-stimulating factor. Curr Opin Hematol.

[34] Zaynagetdinov R, Sherrill TP, Kendall PL, Segal BH, Weller KP, Tighe RM, Blackwell TS (2013). Identification of myeloid cell subsets in murine lungs using flow cytometry. Am J Respir Cell Mol Biol.

[35] Misharin AV, Morales-Nebreda L, Mutlu GM, Budinger GR, Perlman H (2013). Flow cytometric analysis of macrophages and dendritic cell subsets in the mouse lung. Am J Respir Cell Mol Biol.

[36] Elder AC, Gelein R, Oberdorster G, Finkelstein J, Notter R, Wang Z (2004). Efficient depletion of alveolar macrophages using intratracheally inhaled aerosols of liposome-encapsulated clodronate. Exp Lung Res.

[37] Waltl I, Kaufer C, Broer S, Chhatbar C, Ghita L, Gerhauser I, Anjum M, Kalinke U, Loscher W (2018). Macrophage depletion by liposome-encapsulated clodronate suppresses seizures but not hippocampal damage after acute viral encephalitis. Neurobiol Dis.

[38] Wakayama H, Hashimoto N, Matsushita Y, Matsubara K, Yamamoto N, Hasegawa Y, Ueda M, Yamamoto A (2015). Factors secreted from dental pulp stem cells show multifaceted benefits for treating acute lung injury in mice. Cytotherapy.

[39] Pinheiro NM, Santana FP, Almeida RR, Guerreiro M, Martins MA, Caperuto LC, Camara NO, Wensing LA, Prado VF, Tiberio IF (2017). Acute lung injury is reduced by the alpha7nAChR agonist PNU-282987 through changes in the macrophage profile. Faseb j.

[40] Liu C, Chikina M, Deshpande R, Menk AV, Wang T, Tabib T, Brunazzi EA, Vignali KM, Sun M, Stolz DB (2019). Treg cells promote the SREBP1-dependent metabolic fitness of tumor-promoting macrophages via repression of CD8(+) T cell-derived interferon-gamma. Immunity.

[41] Guo Z, Wen Z, Qin A, Zhou Y, Liao Z, Liu Z, Liang Y, Ren T, Xu L (2013). Antisense oligonucleotide treatment enhances the recovery of acute lung injury through IL-10-secreting M2-like macrophage-induced expansion of CD4+ regulatory T cells. J Immunol.

[42] Hamilton TA, Zhao C, Pavicic PG, Datta S (2014). Myeloid colony-stimulating factors as regulators of macrophage polarization. Front Immunol.

[43] Murray PJ, Allen JE, Biswas SK, Fisher EA, Gilroy DW, Goerdt S, Gordon S, Hamilton JA, Ivashkiv LB, Lawrence T (2014). Macrophage activation and polarization: nomenclature and experimental guidelines. Immunity.

[44] Herold S, Mayer K, Lohmeyer J (2011). Acute lung injury: how macrophages orchestrate resolution of inflammation and tissue repair. Front Immunol.

[45] Aggarwal NR, King LS, D'Alessio FR (2014). Diverse macrophage populations mediate acute lung inflammation and resolution. Am J Physiol Lung Cell Mol Physiol.

[46] Yang BY, Deng GY, Zhao RZ, Dai CY, Jiang CY, Wang XJ, Jing YF, Liu XJ, Xia SJ, Han BM (2019). Porous Se@SiO2 nanosphere-coated catheter accelerates prostatic urethra wound healing by modulating macrophage polarization through reactive oxygen species-NF-kappa B pathway inhibition. Acta Biomater.

[47] Shyamsundar M, McAuley DF, Ingram RJ, Gibson DS, O'Kane D, McKeown ST, Edwards A, Taggart C, Elborn JS, Calfee CS (2014). Keratinocyte growth factor promotes epithelial survival and resolution in a human model of lung injury. Am J Resp Crit Care.

[48] Biswas SK, Bist P, Dhillon MK, Kajiji T, Del Fresno C, Yamamoto M, Lopez-Collazo E, Akira S, Tergaonkar V (2007). Role for MyD88-independent, TRIF pathway in lipid A/TLR4-induced endotoxin tolerance. J Immunol.

[49] Kawai T, Adachi O, Ogawa T, Takeda K, Akira S (1999). Unresponsiveness of MyD88-deficient mice to endotoxin. Immunity.

