# Supplementary material for: Manipulation of macrophage polarization by peptide-coated gold nanoparticles and its protective effects on acute lung injury
Source: J Nanobiotechnology. 2020 Feb 26;18:38. doi: 10.1186/s12951-020-00593-7 (PMC7045427; doi:10.1186/s12951-020-00593-7)
Supplement: Supplementary file 1 — Additional file 1. Additional figures describe the zeta-potential of P12, the anti-inflammatory activities of P12 in LPS-induced ALI mice at different doses, the culture procedure and purity of BMDMs, viability of M1 and M2 macrophages by P12 treatment, and the changes of the M2 and M1 macrophage percentage in the lung tissues upon P12 treatment by immunofluorescence imaging. [file 12951_2020_593_MOESM1_ESM.pdf]

Supporting Information for

Manipulation of Macrophage Polarization by Peptide-Coated Gold Nanoparticles and its Protective Effects on Acute Lung Injury

Lu Wang[1,#], Huasheng Zhang[2,#], Liya Sun[3], Wei Gao[1], Ye Xiong[4], Aying Ma[1], Xiali Liu[1], Lei Shen[2,*], Qiang Li[1,4,*], and Hong Yang[1,3,*]

1 Department of Pulmonary and Critical Care Medicine, Shanghai General Hospital, Shanghai Jiaotong University School of Medicine, Shanghai 201620, China

2 Shanghai Institute of Immunology, Shanghai Jiaotong University School of Medicine, Shanghai 200025, China

3 School of Biomedical Engineering, Tianjin Medical University, Tianjin 300070, China

4 Department of Pulmonary and Critical Care Medicine, Shanghai East Hospital, Tongji University, Shanghai 200120, China

# These authors contributed equally to the paper.

* Corresponding author

Prof. Hong Yang *

Email: hongyang@tmu.edu.cn

Prof. Qiang Li *

Email: liqressh@hotmail.com

Prof. Lei Shen*

Email: lshen@shsmu.edu.cn

S1. Supplemental Methods

S2. Supplemental Tables

Table S1.
List of primer sequences for the detection of iNOS, IL-12, Arg 1 and YM-1 mRNA levels by qRT-PCR.

S3. List of Supplementary Figures

Figure S1.
Zeta potentials of bare GNPs and peptide modified GNPs (P12).

Figure S2.
The anti-inflammatory activities of P12 in LPS-induced ALI mice. (a) Schematic illustration of LPS-induced ALI models. P12 (1 μM in 50 μL PBS) or the same volume of PBS was injected into the mouse trachea 2 h before intratracheal LPS (10 mg/kg) challenge. Mice were sacrificed for injury and inflammation analyses 24 h after LPS stimulation. The number of total cells (b) and neutrophils (c) in BALF were analyzed. Representative hematoxylin-eosin (H&E) stained lung sections from (d) PBS+PBS, (e) PBS+LPS, and (f) P12+LPS group. Scale bar represents 100 μm. (g) The total lung injury score was obtained from five independent histological indexes.

Figure S3
P12 reduced lung inflammation in LPS-induced ALI mice at the doses of 250 and 500 nM. The BAL cells were collected for the analysis of the total cell counts (a) and neutrophil counts (b). (c) The IL-10 level in BALF was significantly increased by 500 nM P12 treatment measured by ELISA. N = 9, ns: not significant, *p<0.05, ****p < 0.0001.

Figure S4
The production levels of selected cytokines altered by P12 treatment in ALI mice by Luminex assay. (a-c) BALF; (d-f) serum. N=5, ns: not significant, *p<0.05, ***p < 0.001, ****p < 0.0001.

Figure S5

The culture procedure and purity analysis of BMDMs. (a) The diagram of experimental flow showing the BMDM culture protocol and the cell morphology at various time periods. On day 7, cells were harvested to analyze the purity of mature BMDMs. The mature BMDMs were defined as F4/80$^+$CD11b$^+$ population. The average purity of BMDMs was 95.3% ± 3.2% (mean ± SD). N = 4.

Figure S6

Viability of BMDMs treated with M1 (a) and M2 (b) inducing agents in the absence and presence of various concentrations of P12 measured by MTS assay. 1% triton serves as positive control. n=4, ns: not significant.

Figure S7

The changes of the M2 and M1 macrophage percentage in the lung tissues upon P12 treatment by immunofluorescence imaging. (a) Representative immunofluorescence images of lung tissues co-stained with DAPI, F4/80 (Green) and the M2 marker CD206 (red). (b) Representative immunofluorescence images of lung tissues co-stained with DAPI, F4/80 (green) and the M1 marker iNOS (red). N = 3.

S1. Supplemental Methods

S1.1 Immunofluorescence analyses of lung tissues

The expression of CD206 (M2 macrophage marker) and iNOS (M1 macrophage marker) in lung tissues upon P12 treatment was evaluated by immunofluorescence using standard protocol. The left lungs were dissected from mice without BALF collection, and fixed in 4% paraformaldehyde 24 hours after LPS challenge. The fixed lungs were then embedded in paraffin and cut into 5 μm sections prior to immunofluorescence (IF) assays.

For immunofluorescence staining, the sections were dewaxed and rehydrated, followed by antigen retrieval with citrate buffer solution (10 mM, pH 6.0). After blocking with 10% normal donkey serum (AntGene, CHN), the sections for M2 macrophage identification were incubated with F4/80 antibody (1:200; 70076, Cell Signaling Technology, Inc., USA) and CD206 antibody (1:200; AF2535, R&D Systems, USA) overnight at 4°C, and then incubated with Alexa Fluor®488 donkey anti-rabbit lgG (1:400; A21206, Life technologies, USA) and Alexa Fluor®594 donkey anti-goat lgG (1:400; A11058, Life technologies, USA) for 45 min at 37℃. Since the primary antibodies of F4/80 and iNOS (18985-1-AP, Proteintech Group, Inc., USA) were from the same species, different sections were chosen for M1 macrophages staining. Similarly these sections were first incubated with F4/80 antibody (1:1000) overnight at 4°C after blocking, and then incubated with Goat Anti-Rabbit IgG (H+L) HRP (1:100, S0001, Affinity Biosciences, USA) for 45 min at 37℃ and incubated with FITC dye for conjugation for 15 min. Next, the sections were processed with antigen retrieval and blocking again, and incubated with iNOS antibody (1:400) overnight at 4°C. Afterwards, the sections were incubated with Goat Anti-Rabbit IgG (H+L) HRP for 45 min at 37℃ and then incubated with Cy3 dye for conjugation for 15 min. Finally, these sections were stained with DAPI (Solarbio, Beijing, CHN) for 5 min prior to mounting with Fluoromount-G (SouthernBiotech, USA), and imaged by a Leica TCS SP8 confocal microscope (Wetzlar, Germany) to analyze the fluorescence intensity of CD206 and iNOS and the percentage of positive cells co-stained with F4/80.

S2. Supplementary Tables

Table S1. List of primer sequences for the detection of iNOS, IL-12, Arg 1 and YM1 mRNA levels by qRT-PCR method.

	Sequence, 5'-3'	
**Name**	**Forward**	**Reverse**
M1 markers		
iNOS	TGCCACGGACGAGACGGATAG	CTCTTCAAGCACCTCCAGGAACG
IL-12b	CCTGTGACACGCCTGAAGAAGATG	CTTGTGGAGCAGCAGATGTGAGTG
M2 markers		
ARG1	CTCCAAGCCAAAGTCCTTAGAG	AGGAGCTGTCATTAGGGACATC
YM1	AGAAGGGAGTTTCAAACCTGGT	GTCTTGCTCATGTGTGTAAGTGA

S3. Supplemental Figures

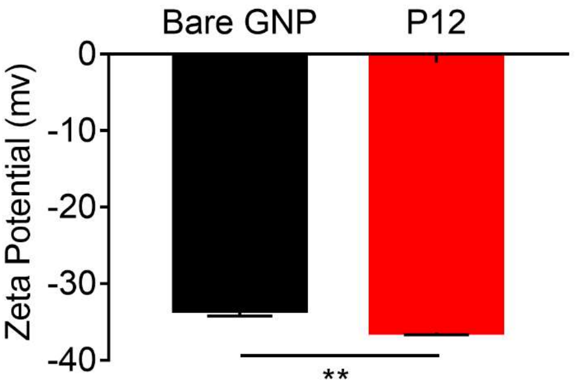

Figure S1. Zeta potentials of the bare GNPs and peptide modified GNPs (P12).

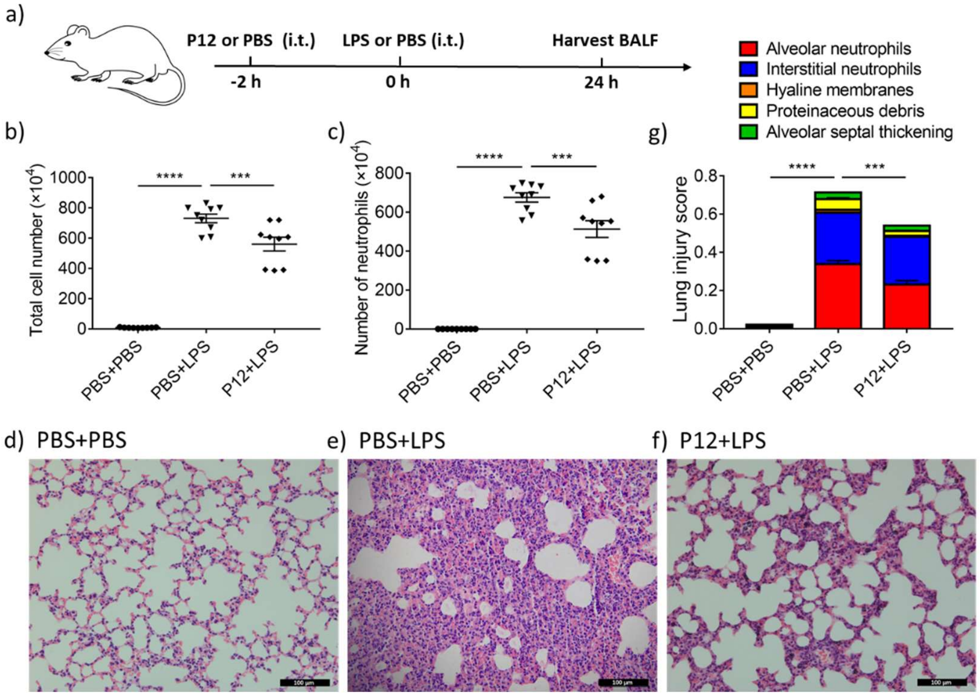

Figure S2. The anti-inflammatory activities of P12 in LPS-induced ALI mice. (a) Schematic illustration of LPS-induced ALI models. P12 (1 μM in 50 μL PBS) or the same volume of PBS was injected into the mouse trachea 2 h before intratracheal LPS (10 mg/kg) challenge. Mice were sacrificed for injury and inflammation analyses 24 h after LPS stimulation. The number of total cells (b) and neutrophils (c) in BALF were analyzed. Representative hematoxylin-eosin (H&E) stained lung sections from (d) PBS+PBS, (e) PBS+LPS, and (f) P12+LPS group. Scale bar represents 100 μm. (g) The total lung injury score was obtained from five independent histological indexes.

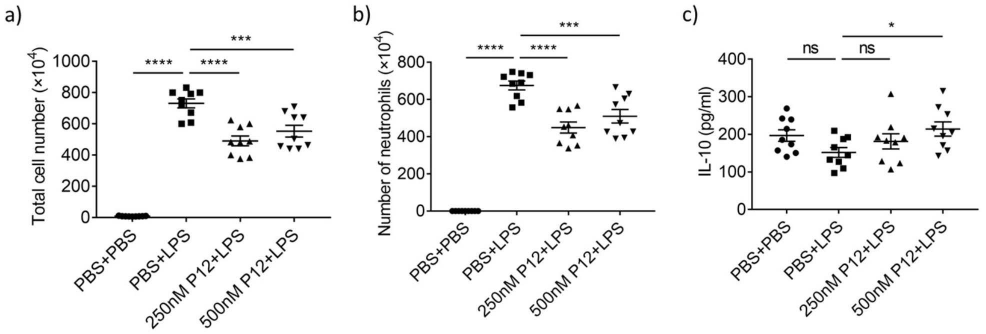

Figure S3. P12 reduced lung inflammation in LPS-induced ALI mice at the doses of 250 and 500 nM. The BAL cells were collected for the analysis of the total cell counts (a) and neutrophil counts (b). (c) The IL-10 level in BALF was significantly increased by 500 nM P12 treatment measured by ELISA. N = 9, ns: not significant, *p<0.05, ****p < 0.0001.

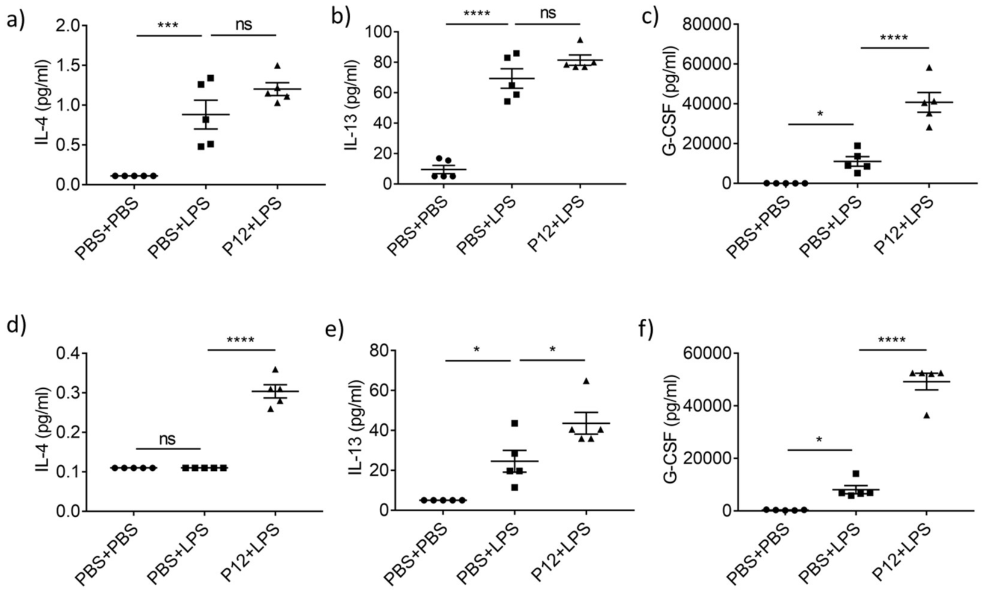

Figure S4. The production levels of selected cytokines altered by P12 treatment in ALI mice by Luminex assay. (a-c) BALF; (d-f) serum. N=5, ns: not significant, *p<0.05, ***p < 0.001, ****p < 0.0001.

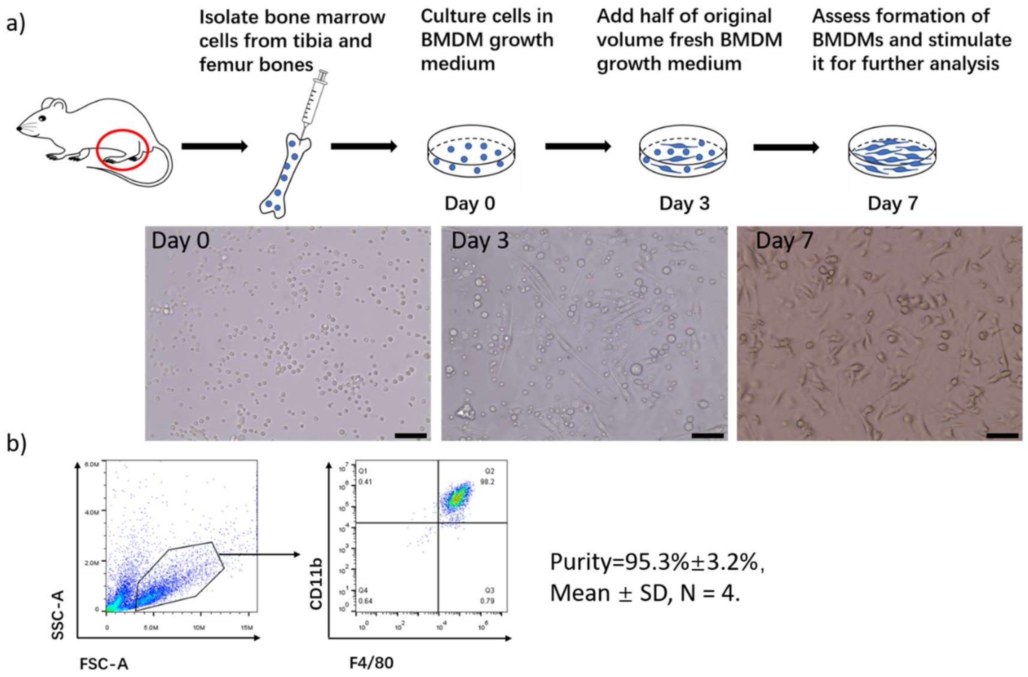

Figure S5. The culture procedure and purity analysis of BMDMs. (a) The diagram of experimental flow showing the BMDM culture protocol and the cell morphology at various time periods. On day 7, cells were harvested to analyze the purity of mature BMDMs. The mature BMDMs were defined as $F4/80^{+}CD11b^{+}$ population. The average purity of BMDMs was 95.3% ± 3.2% (mean ± SD). N = 4.

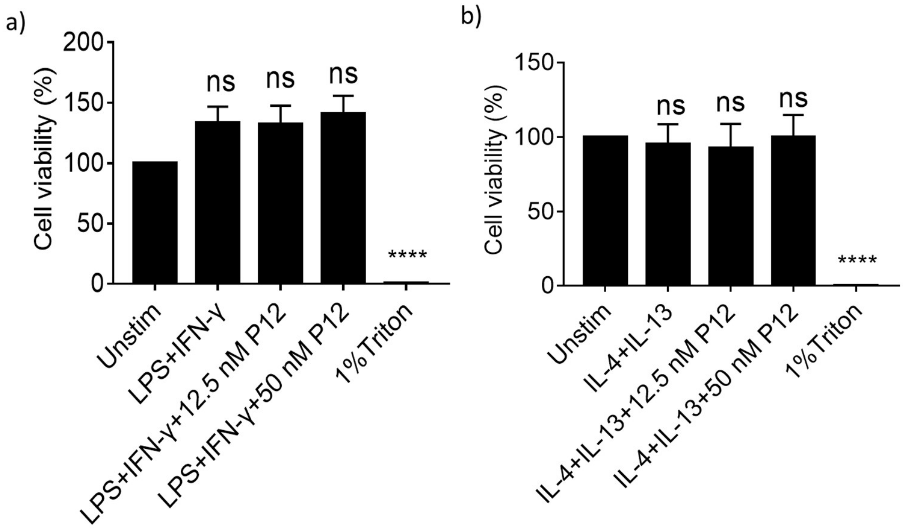

Figure S6. Viability of BMDMs treated with M1 (a) and M2 (b) inducing agents in the absence and presence of various concentrations of P12 measured by MTS assay. Triton (1%) serves as the positive control. n=4, ns: not significant. ****: $p < 0.0001$.

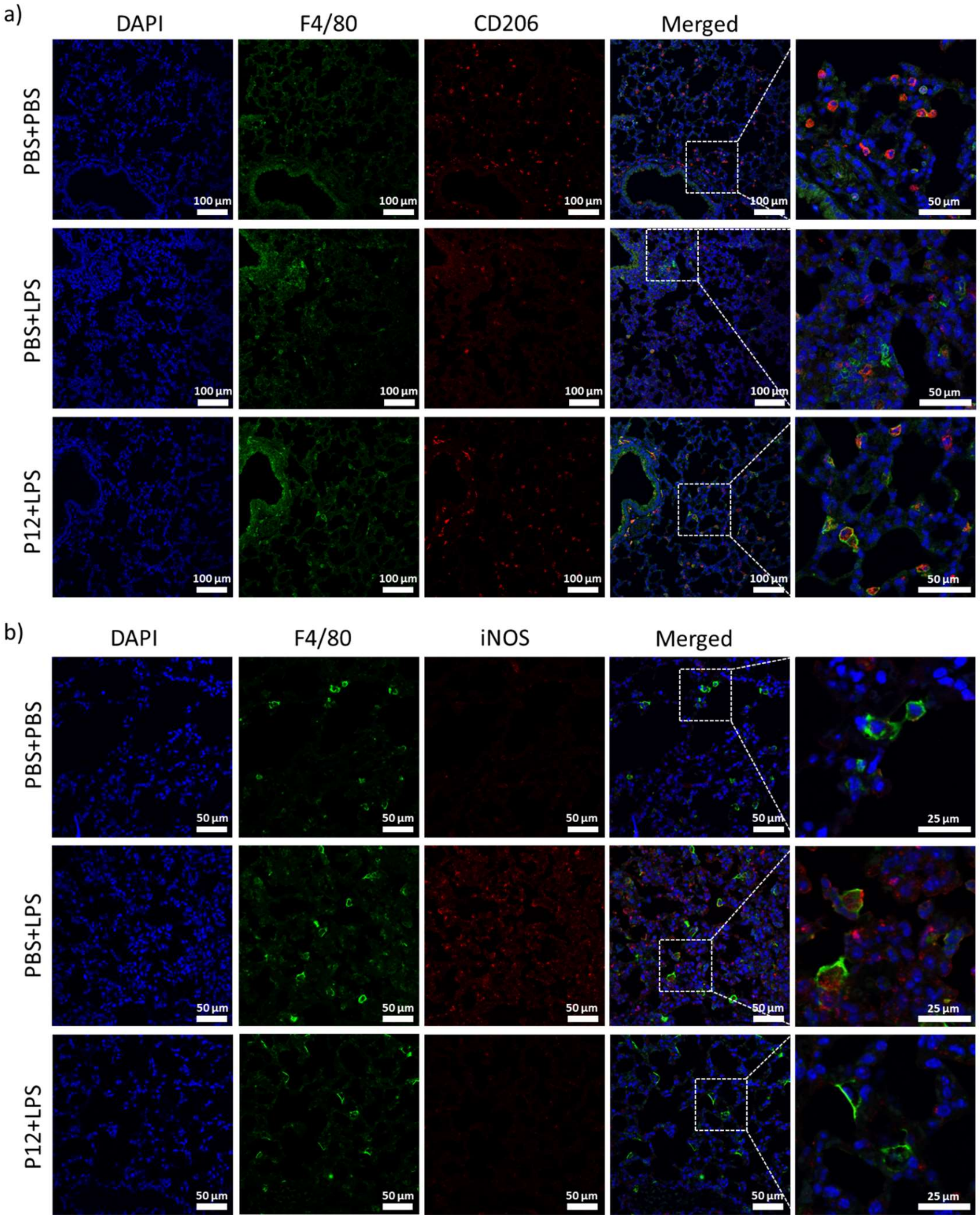

Figure S7. The changes of the M2 and M1 macrophage percentage in the lung tissues upon P12 treatment by immunofluorescence imaging. (a) Representative immunofluorescence images of lung tissues co-stained with DAPI, F4/80 (Green) and the M2 marker CD206 (red). (b) Representative immunofluorescence images of lung tissues co-stained with DAPI, F4/80 (green) and the M1 marker iNOS (red). N = 3.
